# Atlas of ticks (Acari: Argasidae, Ixodidae) in Germany

**DOI:** 10.1007/s10493-021-00619-1

**Published:** 2021-05-03

**Authors:** Franz Rubel, Katharina Brugger, Lidia Chitimia-Dobler, Hans Dautel, Elisabeth Meyer-Kayser, Olaf Kahl

**Affiliations:** 1 Unit for Veterinary Public Health and Epidemiology, University of Veterinary Medicine Vienna, Wien, Austria; 2 Bundeswehr Institute of Microbiology, Munich, Germany; 3 Insect Services GmbH, Berlin, Germany; 4 Thuringian State Office for Consumer Protection, Bad Langensalza, Germany; 5 tick-radar GmbH, Berlin, Germany

**Keywords:** Tick map, Species distribution, Georeferenced data

## Abstract

An updated and increased compilation of georeferenced tick locations in Germany is presented here. This data collection extends the dataset published some years ago by another 1448 new tick locations, 900 locations of which were digitized from literature and 548 locations are published here for the first time. This means that a total of 3492 georeferenced tick locations is now available for Germany. The tick fauna of Germany includes two species of Argasidae in the genera *Argas* and *Carios* and 19 species of Ixodidae in the genera *Dermacentor*, *Haemaphysalis*, and *Ixodes*, altogether 21 tick species. In addition, three species of Ixodidae in the genera *Hyalomma* (each spring imported by migratory birds) and *Rhipicephalus* (occasionally imported by dogs returning from abroad with their owners) are included in the tick atlas. Of these, the georeferenced locations of 23 tick species are depicted in maps. The occurrence of the one remaining tick species, the recently described *Ixodes inopinatus*, is given at the level of the federal states. The most common and widespread tick species is *Ixodes ricinus*, with records in all 16 federal states. With the exception of Hamburg, *Dermacentor reticulatus* was also found in all federal states. The occurrence of the ixodid ticks *Ixodes canisuga*, *Ixodes frontalis*, *Ixodes hexagonus* and *I. inopinatus* were documented in at least 11 federal states each. The two mentioned argasid tick species were also documented in numerous federal states, the pigeon tick *Argas reflexus* in 11 and the bat tick *Carios vespertilionis* in seven federal states. The atlas of ticks in Germany and the underlying digital dataset in the supplement can be used to improve global tick maps or to study the effects of climate change and habitat alteration on the distribution of tick species.

**Supplementary Information:**

The online version supplementary material available at 10.1007/s10493-021-00619-1.

## Introduction

In recent years, the scientific community has become increasingly interested in digital distribution maps of ticks. Georeferenced tick locations, in particular, are needed to study the effects of climate change on the spread of ticks and tick-borne diseases using species distribution models. In Europe, the first digital dataset of the most abundant tick species was compiled by Estrada-Peña et al. (2013). Distribution maps with georeferenced data on the national tick fauna were compiled for Portugal (Santos-Silva et al. 2011), Great Britain (Jameson and Medlock 2011), Belgium (Obsomer et al. 2013), and Germany (Rubel et al. 2014). Nevertheless, there are still major gaps in the knowledge of the distribution of many tick species, including Germany. Existing georeferenced datasets, such as those mapped in the scientific standard book *Ticks of Europe and North Africa* (Estrada-Peña et al. 2017b), are therefore only a first step in describing the occurrence of tick species. For example, the map of the widespread fox tick *Ixodes canisuga* in that book (Sándor 2017) does not show a single location in Germany. The map of the common tick *Ixodes frontalis* (Pfäffle et al. 2017) also shows only one location in Germany. The tick atlas of Germany presented here is intended to provide not only printed maps but also digital data to help close gaps in these and other tick distribution maps.

The first map of the georeferenced ixodid tick locations in Germany was published by Rubel et al. (2014) and contained a collection of 2044 locations of nine tick species. Now, 7 years later, the first tick atlas will be presented here, which includes all 24 tick species found in Germany so far. Therefore, the maps may be considered as an addition to the annotated checklist of the ticks of Germany by Petney et al. (2012, 2015). That checklist also reflects the state of the art of the taxonomic status of the considered tick species. In addition, the checklist provides an overview of the literature on distribution, the hosts, the basic ecology as well as the medical and veterinary importance of the listed tick species (Petney et al. 2012). Similar information is provided in the *The Hard Ticks of the World* (Guglielmone et al. 2014) and in the unfortunately no longer available online atlas *Fauna of Ixodid Ticks of the World* (Kolonin 2009). The host associations of the Hungarian hard tick fauna published by Hornok et al. (2020) can also be applied to ticks occurring in Germany. This information is therefore not repeated here, but reference is made to the relevant sources. An exception is the description of the global distribution of each tick species, which is required as an important additional information for assessing the reliability of the tick locations collected in Germany. For this purpose, information is incorporated from distribution maps recently published, e.g., for *Dermacentor reticulatus* (Rubel et al. 2020) and *Haemaphysalis concinna* (Rubel et al. 2018). Therefore, the focus of this study is on the complete description of the known locations of all tick species in Germany. To achieve this goal, tick locations described in the German-language literature, which are difficult to access for the international scientific community, have been digitized and supplemented by a large number of previously unpublished or new data from the authors.

**Fig. 1:**
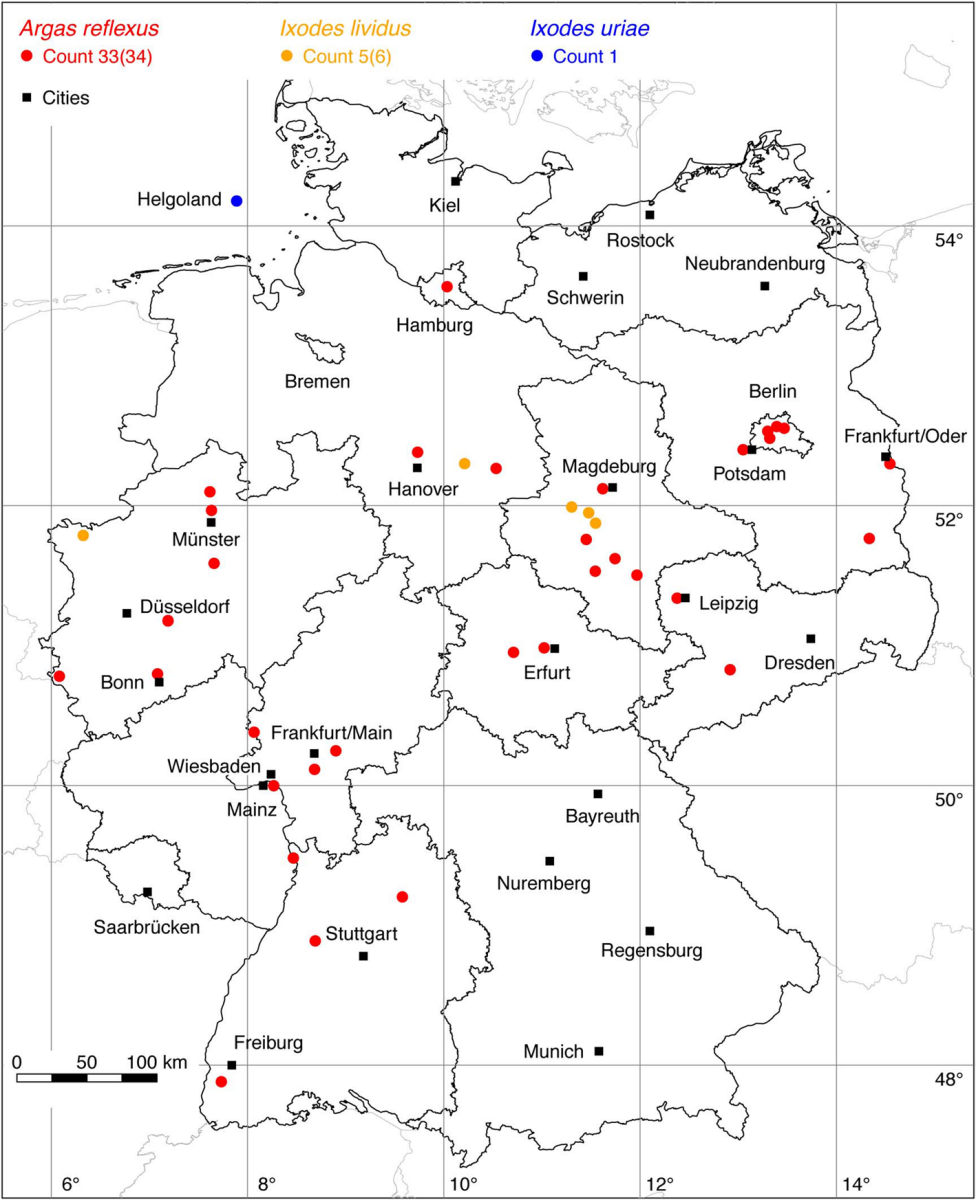
Recorded locations of *Argas reflexus*, *Ixodes lividus* and *Ixodes uriae* in Germany

**Fig. 2:**
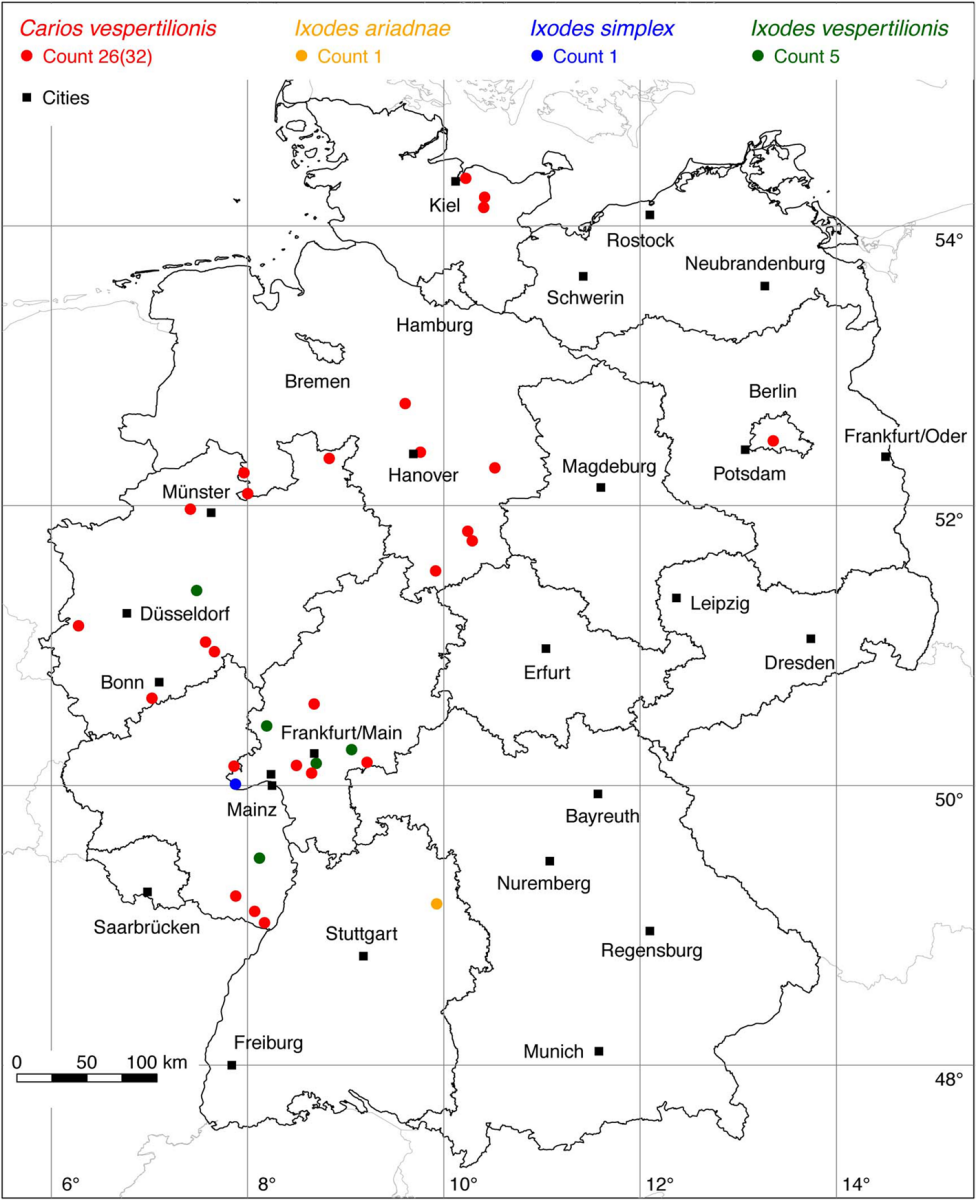
Recorded locations of *Carios vespertilionis*, *Ixodes ariadnae*, *Ixodes simplex* and *Ixodes vespertilionis* in Germany

**Fig. 3:**
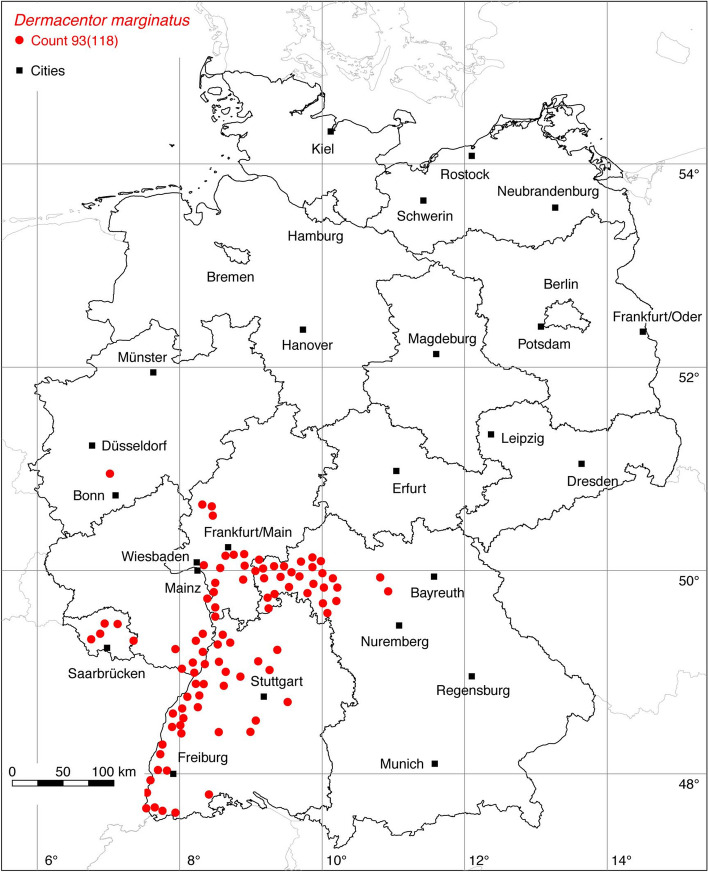
Recorded locations of *Dermacentor marginatus* in Germany

**Fig. 4:**
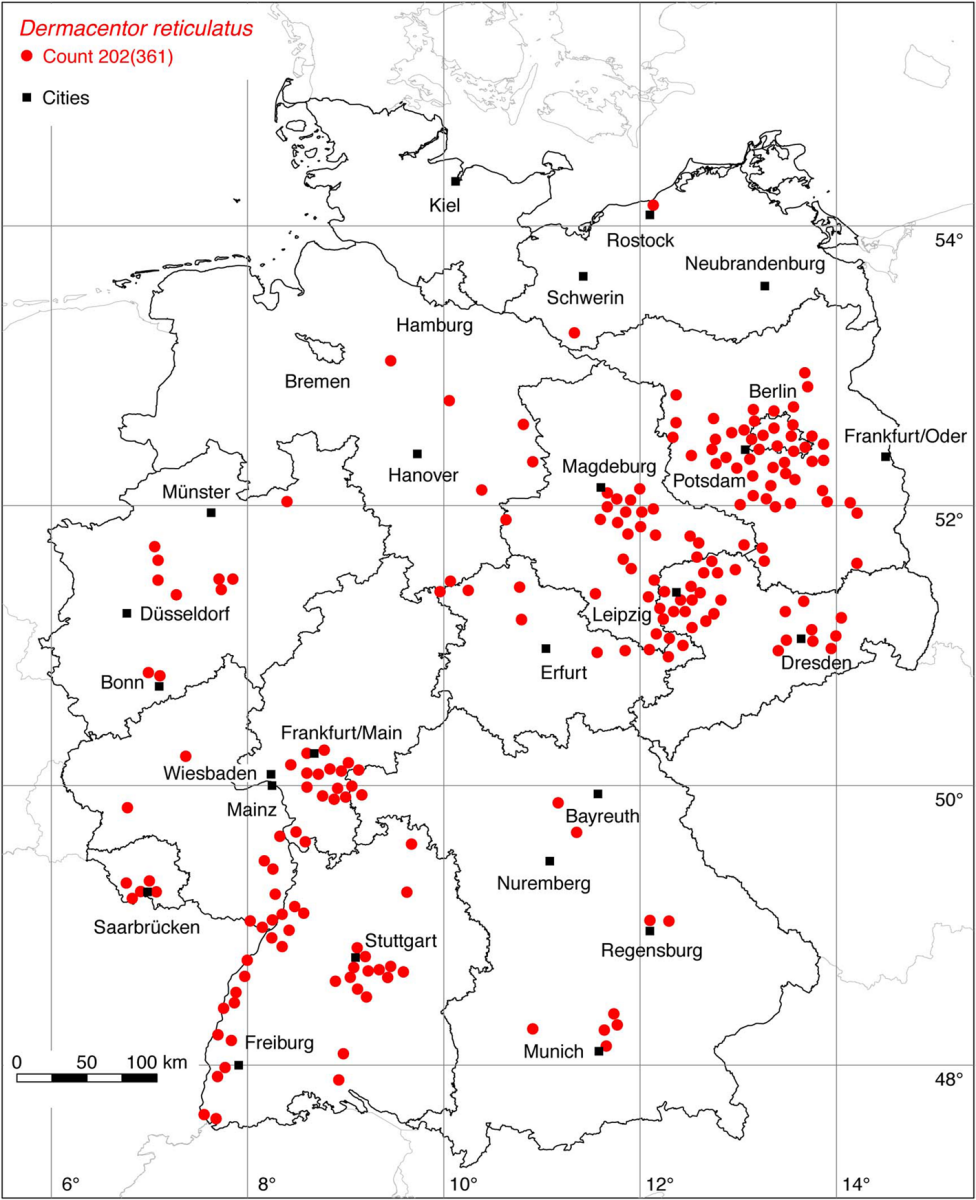
Recorded locations of *Dermacentor reticulatus* in Germany

**Table 1:**
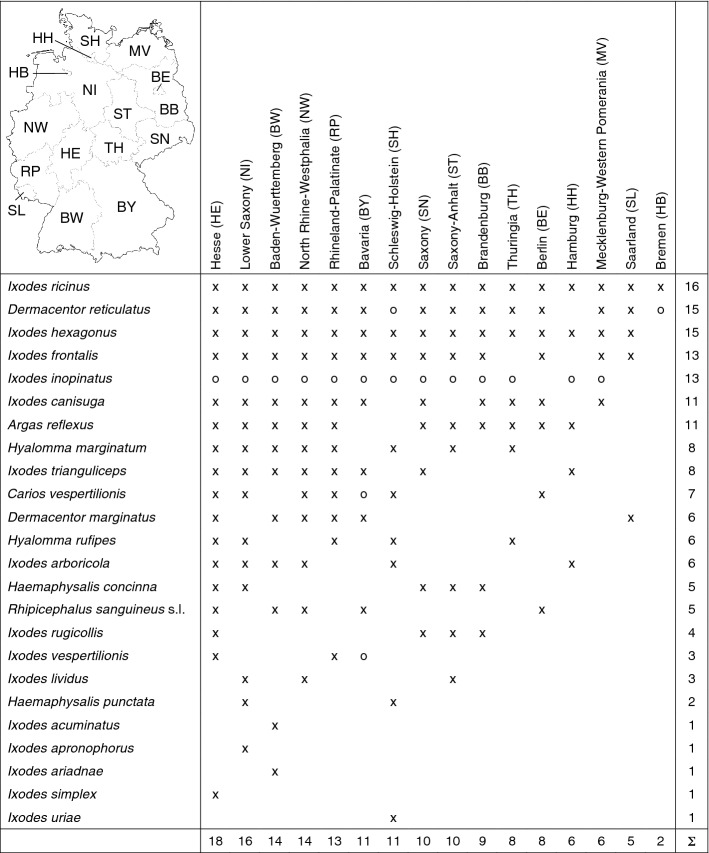
Occurrence of 24 tick species (Acari: Argasidae, Ixodidae) in the 16 German federal states: (×) georeferenced locations of this study, and (o) documented in the scientific literature

## Data and methods

The data used here are based on the 2044 georeferenced tick locations in Germany described by Rubel et al. (2014). These are composed of 1051 compiled tick locations, which have been extended by 776 *Ixodes ricinus* locations from Estrada-Peña et al. (2013) and 217 *I. ricinus* locations from GBIF (2014). Here, this dataset is extended by 1448 new tick locations, 900 locations of which were digitized from literature and 548 new tick locations provided by the authors. Thus, the entire data set used here consists of 3492 georeferenced tick locations. The geographical coordinates of the new tick locations are provided in the supplement together with an indication of their accuracy and the sources. The coordinates are given in decimal degrees with a measure of accuracy divided into high (± 30 m), medium (± 1 km) and low (± 10 km) precision, identical to those previously used by Rubel et al. (2014, 2018, 2020).

**Fig. 5:**
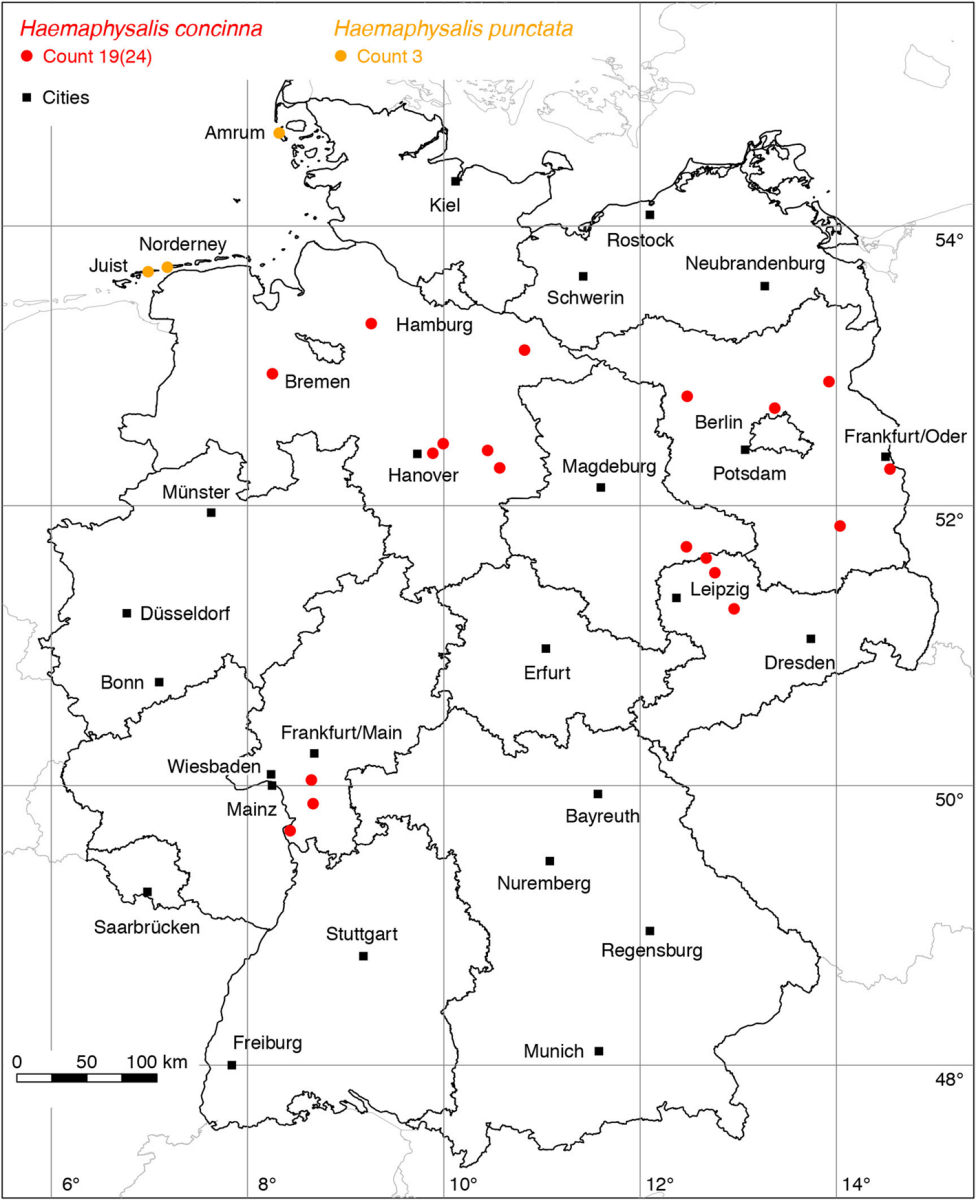
Recorded locations of *Haemaphysalis concinna* and *Haemaphysalis punctata* in Germany

**Fig. 6:**
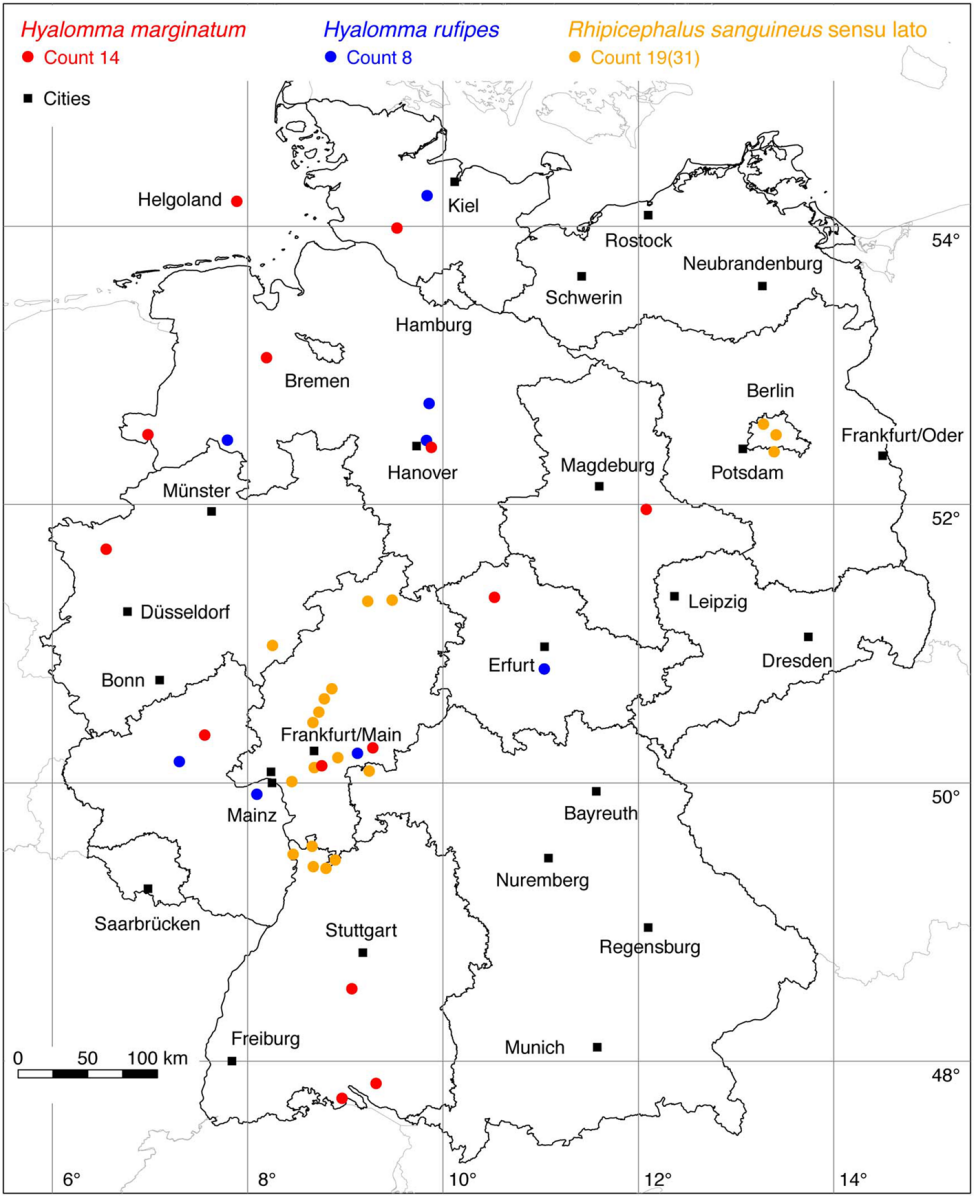
Recorded locations of *Hyalomma marginatum*, *Hyalomma rufipes* and *Rhipicephalus sanguineus* in Germany. These species are not endemic in Germany, but are continuously introduced

**Fig. 7:**
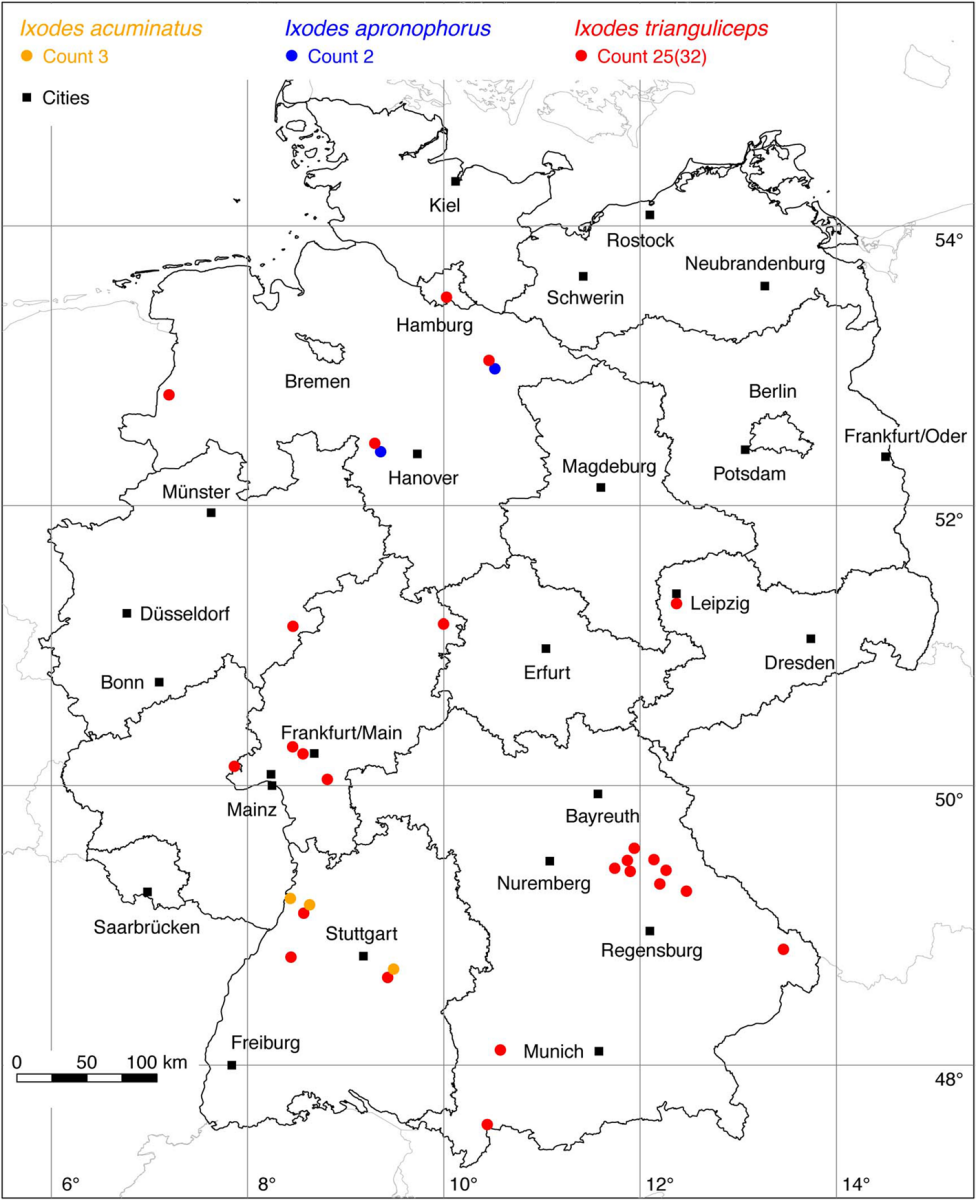
Recorded locations of *Ixodes acuminatus*, *Ixodes apronophorus* and *Ixodes trianguliceps* in Germany

**Fig. 8:**
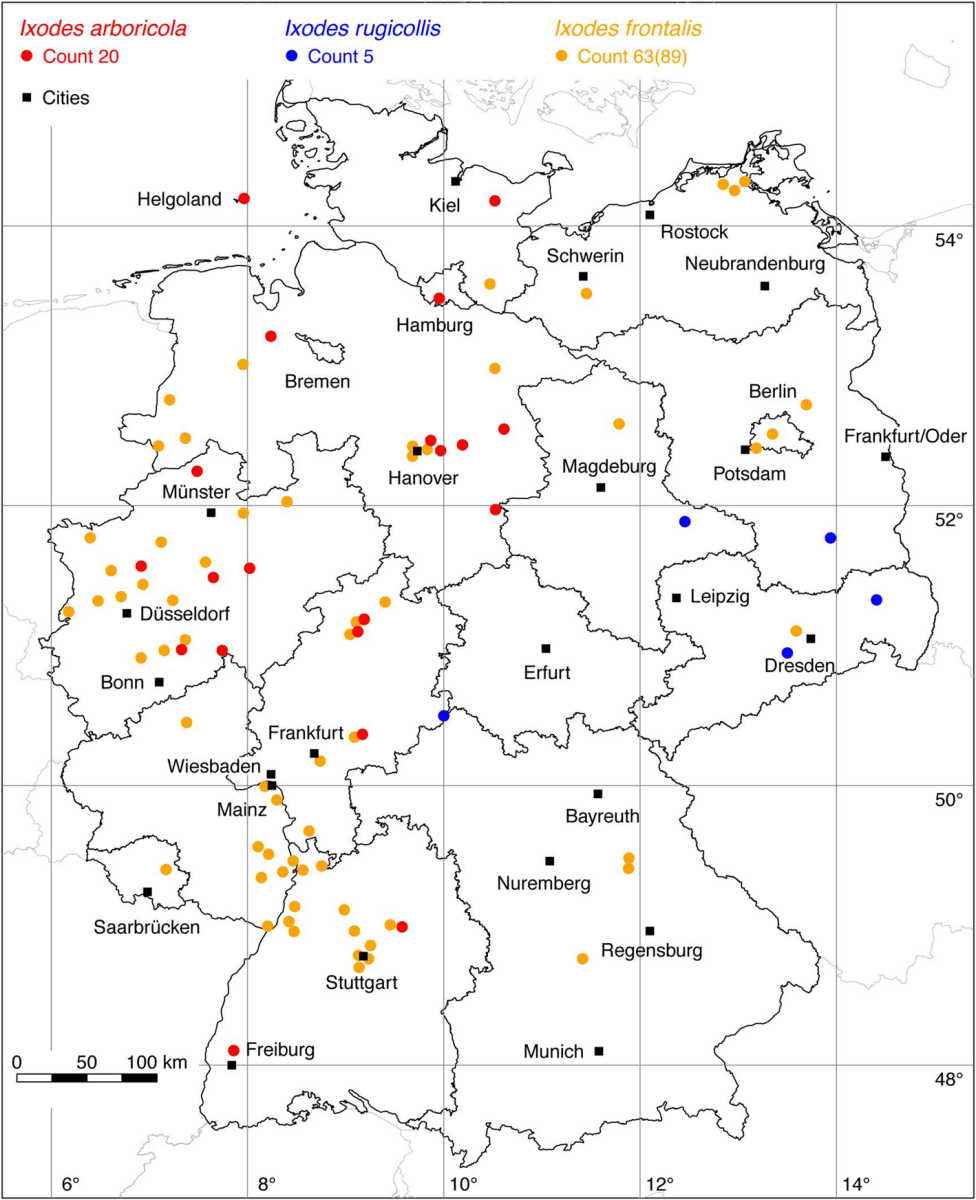
Recorded locations of *Ixodes arboricola*, *Ixodes frontalis* and *Ixodes rugicollis* in Germany

**Fig. 9:**
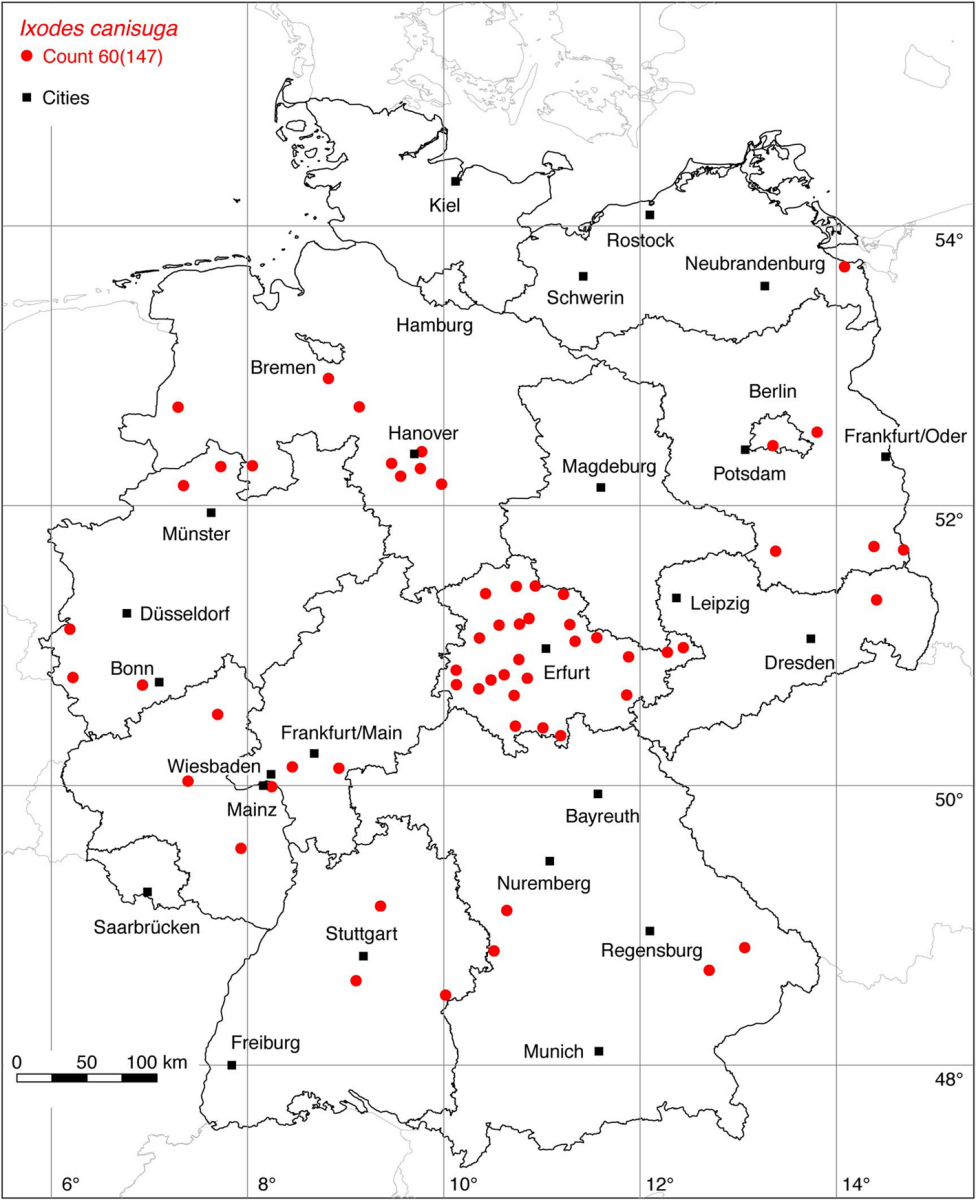
Recorded locations of *Ixodes canisuga* in Germany

**Fig. 10:**
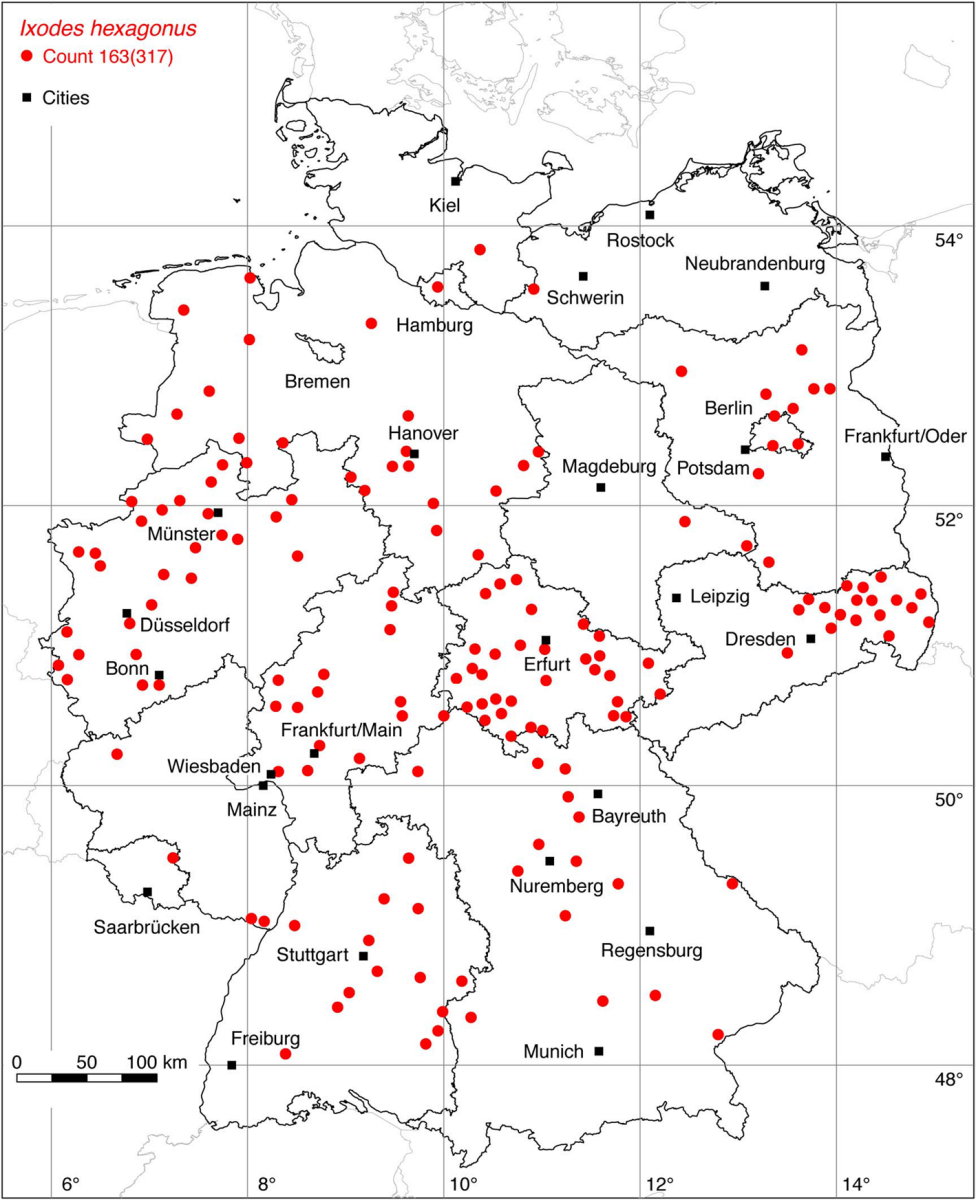
Recorded locations of *Ixodes hexagonus* in Germany

**Fig. 11:**
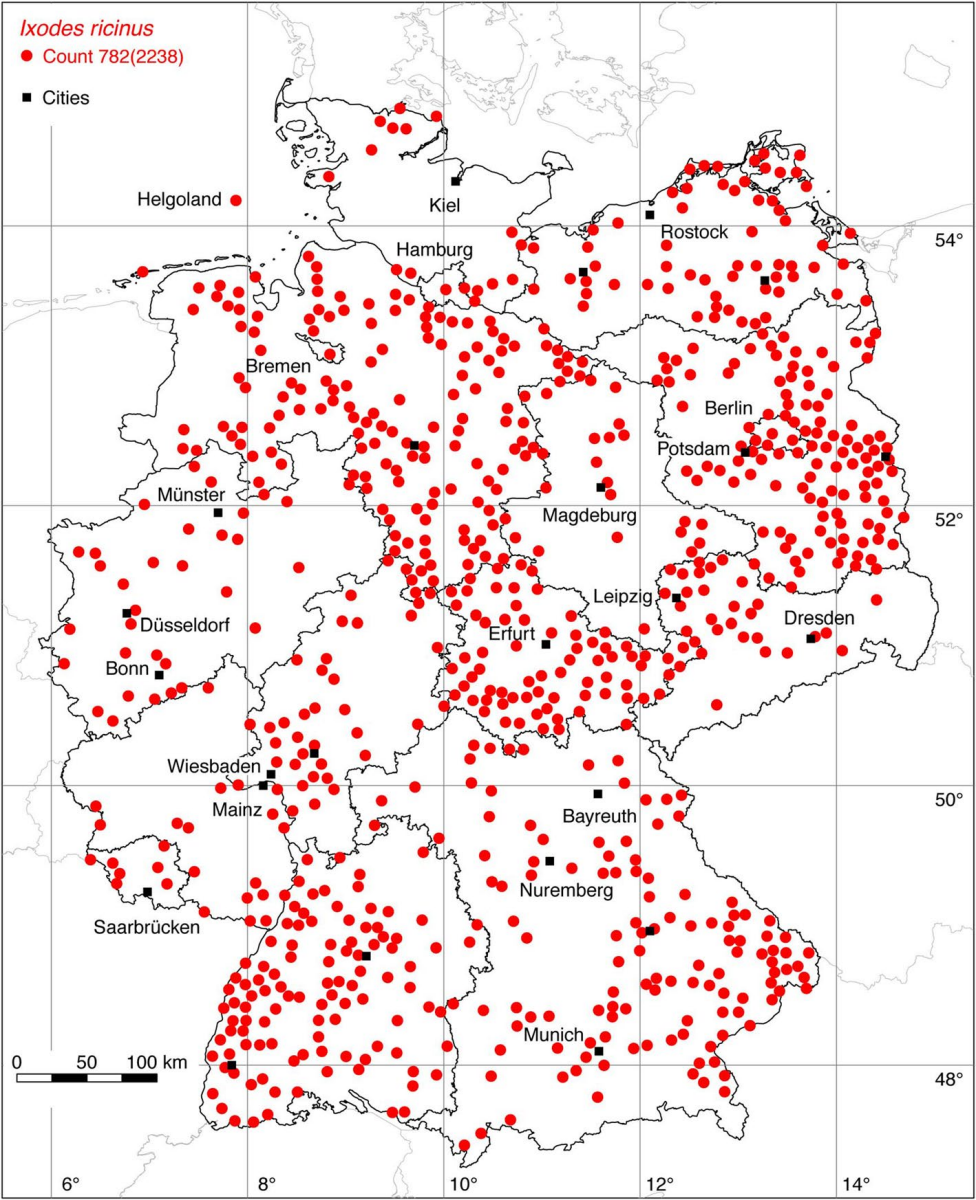
Recorded locations of the *Ixodes ricinus/inopinatus* species complex in Germany

The tick locations are mapped using R, a language and environment for statistical computing (R Development Core Team 2019). However, they are not evenly distributed across Germany. For example, the areas around the large cities Berlin, Hanover, Leipzig and Stuttgart are particularly well covered with data, since research groups have been dealing with ticks and tick-borne diseases here for a long time. Large-scale studies on the tick infestation of wildlife, such as those on red foxes (*Vulpes vulpes*) in Thuringia (Meyer-Kayser et al. 2012) and on the Eurasian otter (*Lutra lutra*) in Upper Lusatia (Christian 2012), also lead to an unusual clustering of tick locations. In order to achieve a more realistic representation of the distribution of the individual tick species, these clusters were reduced in a two-stage process. First, the tick locations of the two studies mentioned were reduced with the help of a random selection. For example, from the study by Meyer-Kayser et al. (2012), only 25 of the 106 available *I. canisuga* locations were shown in the corresponding map. The following numbers of tick locations that have been selected to avoid large-scale clusters caused by data from this study were used: 25(106) for *I. canisuga*, 30(149) for *I. hexagonus*, and 50(195) for *I. ricinus*. Similarly, only 20(34) tick locations from the study by Christian (2012) were used for *I. hexagonus*. To further avoid local clustering and associated sampling biases in the dataset a spatial thinning algorithm was applied (Aiello-Lammens et al. 2015). The ‘thin’ function in the spThin R package provided by Aiello-Lammens et al. (2019) uses a randomization approach and returns a dataset with the maximum number of locations for a given thinning distance, here 8 km. The maps for the individual tick species (Figs. 1, 2, 3, 4, 5, 6, 7, 8, 9, 10, 11) therefore not only show the number of tick locations mapped, but also the total number of available tick locations in brackets.

Tick species, for which only a few locations are known, are grouped according to their host preferences as proposed by Hornok et al. (2020). For example, the bat ticks *Carios vespertilionis*, *Ixodes ariadnae*, *Ixodes simplex*, and *Ixodes vespertilionis* are depicted in the same map.

## Results and discussion

The tick fauna of Germany includes two species of Argasidae in the genera *Argas* and *Carios* and 19 species of Ixodidae in the genera *Dermacentor*, *Haemaphysalis*, and *Ixodes*, altogether 21 tick species. In addition, three species of Ixodidae in the genera *Hyalomma* and *Rhipicephalus* were included in the atlas of ticks. Engorged nymphs of *Hyalomma marginatum* and *Hyalomma rufipes* are imported by migratory birds each spring and unfed or feeding adults have been found regularly throughout Germany since 2018 (Chitimia-Dobler et al. 2019). The tick *Rhipicephalus sanguineus* sensu lato has been occasionally imported by dogs returning with their owners from the Mediterranean and other countries with a subtropical or tropical climate (Gothe and Hamel 1973).

The outcome of this study are geographical maps that depict the occurrence of all tick species that have so far been reported in Germany. The apparently widespread *I. inopinatus* (Estrada-Peña et al. 2014) is an exception. No map has been compiled for *I. inopinatus*, which is combined with *I. ricinus* to form the so-called *I. ricinus/inopinatus* complex here. This allows historical records of *I. ricinus* ticks to be mapped, which might occasionally include specimens that would now be identified as *I. inopinatus*. Although there is also quite a number of records of morphologically determined *I. inopinatus* in many parts of Germany (Chitimia-Dobler et al. 2018), more work is needed to obtain reliable identification of putative *I. inopinatus*. A contribution to this is the molecular identification of *I. inopinatus* by Hauck et al. (2019).

Each tick species is presented below with a brief summary of its global distribution and the numbers of georeferenced locations in Germany compiled for this study. If the ticks were collected from hosts, these are also mentioned. Concerning any further details on the biology and ecology of the mentioned species, readers are referred to the excellent reviews by Petney et al. (2012, 2015).

### *Argas (Argas) reflexus* (Fabricius)

The pigeon tick, *A. reflexus* can be found from Portugal to the North Caucasus, in Europe up to 55° N (Dautel et al. 1991). *Argas reflexus* generally occurs in or close to the nests or resting places of their hosts (Dautel et al. 1999). The principal hosts of *A. reflexus* are domestic pigeons (*Columba livia domestica*) and rock pigeons (*C. livia livia*), which are a characteristic component of many European towns. As a consequence this tick has long been known as a common pest in pigeon coops, firstly described in Germany in 1860 (Metz 1911). Because this tick can infest humans, especially when the natural host is not available for some years, it was an occasional cause of medical problems in the 1980s and 1990s (Fuhrmans and Manske 1983). Records of *A. reflexus*—infested buildings in Berlin districts in the 1990s have been documented by Dautel et al. (1999). However, it can be assumed that the abundance of *A. reflexus* in German towns has decreased over the past two decades because many old houses were replaced or renovated. A few other bird species have also been found to be infested, for example a barn owl (*Tyto alba*) in Baden-Wuerttemberg (Graef 2000). The following number of locations were taken for the *A. reflexus* distribution map: 3 (Mayer and Madel 1950), 1 (Fuhrmans and Manske 1983), 26 (Dautel et al. 1991), 1 (Graef 2000), 1 OK (from 1978), 2 HD (from 2010 and 2014). Here as in the following text, the capital letters refer to the initials of the responsible authors. A total of 33 out of 34 known *A. reflexus* observations is depicted in Fig. 1.

### *Carios (Carios) vespertilionis* (Latreille)

The short-legged bat tick *C. vespertilionis* (also known as *Argas vespertilionis*) is widely distributed in the Old World from the Palaearctic to South Africa (Hoogstraal 1956) south of 60° N latitude (Haitlinger and Walter 1997). The taxonomical status of this tick species like that of many other argasids is not certain. It is unclear whether it is a member of the Argasinae or the Ornithodorinae, and there may exist two or even more cryptic species (Hornok et al. 2017). Mans et al. (2021) presented strong evidence to place it into the genus *Carios* as part of the Ornithodorinae and we herein follow this suggestion. Main hosts are cave-dwelling, insectivorous bats (Petney et al. 2017). The main host in Germany is the common pipistrelle (*Pipistrellus pipistrellus*), but the tick has also been found on 10 other bat species in Bavaria that are believed to be casual hosts (Rupp et al. 2004). The description of an exceptionally strong infestation of a northern bat (*Eptesicus nilssonii*) with *C. vespertilionis* is noteworthy (Walter and Rackow 2007). The following locations have been documented: 8 (Walter and Kock 1985), 6 (Walter 1985), 16 (Haitlinger and Walter 1997), 1 (Walter and Rackow 2007), 1 (Scheffler and Hiller 2010). A total of 26 out of 32 known locations of the soft tick *C. vespertilionis* is mapped in Fig. 2.

### *Dermacentor marginatus* (Sulzer)

The ornate sheep tick *D. marginatus* is mainly found in Mediterranean countries (Rubel et al. 2016) as well as in the Middle East and in countries of the former Soviet Union (Kulik and Vinokurova 1982). In China, its occurrence in the Uighur autonomous region of Xinjiang is confirmed (Teng 1982), although locations further east have also been described (Chen et al. 2010). It follows that the global distribution of *D. marginatus* extends from the Atlantic coast of Portugal to Western Siberia and Xinjiang, 9° W–92° E. In the north-south direction *D. marginatus* is distributed within the latitude belt of 33–58° N, while it occurs in Western and Central Europe only up to 51° N. In Germany, the tick is only found in the Rhine valley and adjacent areas, where it is warmer than in the rest of the country, which is why some Mediterranean animal and plant communities find good living conditions there (Walter et al. 2016). The 77 existing locations from Rubel et al. (2014) have been supplemented by the following number of locations: 5 (Walter et al. 1986), 8 (Pluta et al. 2010; Pluta 2011), 2 (Gilgenast 2013), 1 (Dries 2013), 22 (Kimmig 2010), and 3 OK. A total of 93 out of 118 known locations is mapped in Fig. 3.

### *Dermacentor reticulatus* (Fabricius)

The global distribution of the ornate dog tick *D. reticulatus*, also known as the marsh tick in Germany, was recently mapped by Rubel et al. (2020). Accordingly, its distribution extends from the Atlantic coast of Portugal to Western Siberia, 9° W–88° E, within the latitude range 34–60° N. In Germany, *D. reticulatus* is particularly widespread and common in the eastern federal states of Brandenburg, Saxonia, Saxonia-Anhalt and Berlin. Further locations cluster in the Rhine Valley and in the Saarland. Occasionally, the tick has also been found in other parts of Germany. The sources of this data compilation are documented in Rubel et al. (2020) and have been supplemented by the following number of locations: 199 (Nauke 2007), 11 (Silaghi et al. 2020) and 10 OK. The northernmost German location of *D. reticulatus* flagged from vegetation was reported in 2020 by OK at the Baltic Sea coast in the port of Rostock (Rubel et al. 2020). An even more northern location (but unconfirmed as yet) on the island of Sylt is also known from a citizen science project (Drehmann et al. 2020). This might be a further indication that *D. reticulatus* has expanded its distribution to the north in recent decades. A total of 202 out of 361 known locations is mapped in Fig. 4.

### *Haemaphysalis (Haemaphysalis) concinna* Koch

The global distribution of *Ha. concinna*, the relict tick, was recently mapped by Rubel et al. (2018). Accordingly, its distribution extends from the Spanish Atlantic coast to Kamchatka (Russia), 6° W–159° E. In Europe, *Ha. concinna* occurs within the latitude belt of 40–56° N. It colonizes forest steppes and wet steppe habitats. In Germany, *Ha. concinna* is frequently reported together with *I. ricinus* and *D. reticulatus* (Kahl et al. 1992), but its occurrence is patchy. The 8 locations compiled by Rubel et al. (2014) have been supplemented by the following numbers of locations: 1 Schulze (1923), 7 (Walter et al. 1986), 2 (Cornely and Schultz 1992), 4 (Talaska et al. 2010), 1 LCD, and 1 OK. A total of 19 out of 24 known locations is mapped in Fig. 4.

### *Haemaphysalis (Aboimisalis) punctata* Canestrini and Fanzago

The global distribution of *Ha. punctata*, also known as the red sheep tick, extends over the entire Mediterranean area of Europe and Northern Africa (Estrada-Peña et al. 2013) to Russia (Kolonin 2009) and China (Chen et al. 2010). Although the tick also occurs in more northern latitudes such as the south of Great Britain with its mild climate caused by the Gulf Stream, it has not been observed in Germany for a long time. The only reports of *Ha. punctata* in Germany were documented by Liebisch and Rahman (1976) on the North Frisian island Amrum and the East Frisian islands Norderney and Juist. The tick was found on cattle and sheep pastures near the coast on animals and on the vegetation. Recent *Ha. punctata* reports from the Dutch coast and from the nearby Dutch island of Texel (Hofmeester et al. 2016) confirm the reliability of the three known locations in the northwest of Germany shown in Fig. 5.

### *Hyalomma (Euhyalomma) marginatum* Koch

The global distribution of *Hy. marginatum*, with the junior synonym *Hy. plumbeum* (Petney et al. 2012), extends over the Mediterranean area of Europe and Northern Africa to Western Siberia, 9° W–88° E (Kolonin 2009). Estrada-Peña et al. (2013) determines the northern distribution limit of *Hy. marginatum* south of the European Alps at about 45° N latitude. From there the Mediterranean *Hyalomma* tick, is continuously introduced to Germany by migratory birds and has been observed more and more frequently in recent years. It is unclear at present whether or not *Hy. marginatum* has succeeded in establishing itself in parts of Germany. However, the increased occurrence of adult *Hy. marginatum* seems to be caused by three extraordinarily warm growing seasons in Germany, which allowed many ticks to develop from the engorged nymph to the adult stage. For the first time in Germany, nymphs were described on migratory birds from the island of Helgoland (Walter et al. 1979b). Adult ticks were mostly found in horse stables or on horses (Chitimia-Dobler et al. 2019), the most cared-for large mammals in Germany. The following locations have been documented: 1 Walter et al. (1979b), 1 (Kampen et al. 2007), 1 (Rumer et al. 2011), 1 (Oehme et al. 2017), 9 Chitimia-Dobler et al. (2019), 1 EMK. A total of 14 known locations is depicted in Fig. 6).

### *Hyalomma (Euhyalomma) rufipes* Koch

The hairy or coarse bont-legged *Hyalomma* tick, *Hy. rufipes*, was considered a subspecies of *Hy. marginatum* until it was accepted as a valid species. It is the most widespread *Hyalomma* species in Africa, but is also present in many Mediterranean countries, Iran, Iraq, Kazakhstan, Russia, Saudi Arabia, Tajikistan, Turkmenistan, Ukraine, and Uzbekistan (Apanaskevich and Horak 2008). As already described for the tick *Hy. marginatum*, *Hy. rufipes* is regularly introduced by migratory birds to Germany and other countries north of their natural distribution range (Hubálek et al. 2020). Recently, it was hypothesized that adult *H. rufipes* might overwinter under local climatic conditions in Central Europe (Rudolf et al. 2021). Adult ticks, especially, which infest horses and cattle have been found there. The first *Hy. rufipes* documented in Germany was removed from a horse in Ober-Olm, near the city of Mainz, Rhineland-Palatinate (Chitimia-Dobler et al. 2016). A further location of two *Hy. rufipes* ticks collected from a horse in Thuringia was provided by EMK. Together with the locations described by Chitimia-Dobler et al. (2019), a total of eight known locations is depicted in Fig. 6.

### *Ixodes (Ixodes) acuminatus* Neumann

This tick species has a wide Palaearctic distribution, mainly in broad-leaved and mixed forests of the temperate climate zone (Guglielmone et al. 2014). In Germany, three locations in Baden-Wuerttemberg have been reported by Petney et al. (2013), who found *I. acuminatus* on small and medium-sized mammals (Fig. 7).

### *Ixodes (Ixodes) apronophorus* Schulze

The tick is known from Eastern European forest steppes, the Carpathian mountain forests, Scandinavia, and the Russian taiga (Guglielmone et al. 2014). In Germany, two locations have been reported. In the nature reserve Hagenburger Moor in Lower Saxony *I. apronophorus* was found on small and medium-sized mammals such as the Eurasian water shrew (*Neomys fodiens*), the common vole (*Microtus arvalis*), the field vole (*Microtus agrestis*), the Eurasian harvest mouse (*Micromys minutus*), the striped field mouse (*Apodemus agrarius*), the yellow-necked mouse (*Apodemus flavicollis*), and the brown rat (*Rattus norvegicus*) (Walter 1980). Further findings from Lower Saxony were reported from the administrative district Lüneburg (Olbrich and Liebisch 1991). Both locations are depicted in Fig. 7.

### *Ixodes (Pholeoixodes) arboricola* Schulze and Schlottke

The bird tick *I. arboricola* occurs in Northern Africa, Europe, Russia and China (Guglielmone et al. 2014). It was mainly found on cave breeders and it appears that it is widespread and moderately common in Germany. Walter et al. (1986) found the tick on birds such as the European pied flycatcher (*Ficudela hypoleuca*), the collared flycatcher (*F. albicollis*), the little owl (*Athene noctua*), and the widespread peregrine falcon (*Falco peregrinus*). A total of 15 locations has been digitized from Hudde and Walter (1988), which includes the locations published by Walter et al. (1986). These data are supplemented by the following locations: 1 (Walter et al. 1979a), 1 (Walter 1985) and 3 LCD. A total of 20 known locations is depicted in Fig. 8.

### *Ixodes ariadnae* Hornok et al.

The recently discovered *I. ariadnae* is one of four species of ticks known to mainly infest bats in Germany. This tick was recorded from only one place in Germany where it was removed from a greater mouse-eared bat (*Myotis myotis*), hibernating in a natural cave at the north rim of the river Bühler in Baden-Wuerttemberg (Hornok et al. 2015). The up to now single location of *I. ariadnae* in Germany is shown together with the reported locations of the three other bat ticks *I. simplex*, *I. vespertilionis*, and *C. vespertilionis* in Fig. 2.

### *Ixodes (Pholeoixodes) canisuga* Johnston

In Germany, *I. canisuga* frequently infests red foxes (*Vulpes vulpes*), which is why it is also known as the fox tick. According to Kolonin (2009), the tick is distributed from the Spanish Pyrenees to the east of China between 4.5°W–144.0°E and 32.5–58.5°N. There are records from almost all European countries. In Asia, locations are known from Russia, Iran, Afghanistan, India (Kashmir), and China. The tick is native to all of Germany, although an extensive field study was carried out only in Thuringia (Meyer-Kayser et al. 2011, 2012). The following numbers of locations have been compiled for this study: 3 (Walter et al. 1986), 23 (Liebisch and Walter 1986), 12 (Cornely and Schultz 1992), 1 (Christian 2010), 2 (Kretschmar 2016). This dataset is supplemented by so far unpublished georeferenced coordinates from Meyer-Kayser et al. (2012), comprising the following number of locations: 106 EMK. A total of 60 out of 147 known locations is depicted in Fig. 9.

### *Ixodes (Trichotoixodes) frontalis* (Panzer)

The tick *I. frontalis* infests avian hosts mainly in Southern and Central Europe, Northern Africa and the western part of Russia (Kolonin 2009) and is frequently disseminated by migratory birds (Toma et al. 2021). Sporadic records are also available from migratory birds in Scandinavia, but it is uncertain whether *I. frontalis* is established there (Pfäffle et al. 2017). In Germany, the following number of locations have been recorded: 1 (Schorn et al. 2011), 65 (Drehmann et al. 2019). Additionally, unpublished georeferenced coordinates from *I. frontalis* recently flagged from the vegetation by two of the authors, including larvae flagged in and close to Berlin (Kahl et al. 2019), supplement the data with the following number of locations: 6 LCD, 17 OK. A total of 63 out of 89 known locations is depicted in Fig. 8.

### *Ixodes (Pholeoixodes) hexagonus* Leach

The occurrence of *I. hexagonus*, often referred to as the hedgehog tick, is limited to Europe (Kolonin 2009) and the neighboring Turkey (Bursali et al. 2012). Documented locations range from Portugal, Northern Spain and Great Britain to Central Europe, the Balkans and Turkey. Accordingly, the distribution area ranges from 9.5° W to 41° E, within the latitude belt 37–59° N. For Germany, the following numbers of georeferenced locations have been compiled: 74 (Liebisch and Walter 1986), 10 (Cornely and Schultz 1992), 4 (Christian 2002, 2010), 34 Christian (2012), 4 Speck et al. (2013), 1 (Faulde et al. 2014), 3 (Schreiber et al. 2014), 25 (Kretschmar 2016). Most studies, such as those by Pfäffle et al. (2011) and Meyer-Kayser et al. (2012), reported *I. hexagonus* on European hedgehogs (*Erinaceus europaeus, E. roumanicus*) and red foxes (*Vulpes vulpes*). Records from dogs and cats are also available (Geurden et al. 2018). Noteworthy is the study by Christian (2012), in which *I. hexagonus* was collected in Upper Lusatia, Saxony, from the Eurasian otter (*Lutra lutra*), and the study by Kretschmar (2016), in which *I. hexagonus* was collected in the northwest of Germany from the European polecat (*Mustela putorius*). This dataset is supplemented by up to now unpublished locations of the authors, comprising the following number of locations: 9 HD, 195 EMK, 4 LCD. A total of 163 out of 317 known locations is depicted in Fig. 10.

### *Ixodes (Ixodes) inopinatus* Estrada-Peña, Nava and Petney

This recently described tick species was reported from Portugal, Spain, Germany, Austria, Romania, Morocco and Tunisia (Estrada-Peña 2017; Chitimia-Dobler et al. 2018; Younsi et al. 2020). Its exact global distribution has yet to be determined. Before *I. inopinatus* was described as a new species, it was identified as *I. ricinus*. Molecular identification of tick species collected in Hanover (Lower-Saxony) and Hamburg demonstrated that approximately 3–4% of all ticks previously identified as *I. ricinus* may actually be *I. inopinatus* (Hauck et al. 2019). In Bavaria, this proportion (adults plus nymphs) was 1–7%, with over 15% of the nymphs at two locations being identified as *I. inopinatus* (Chitimia-Dobler et al. 2018). A field study currently underway with the participation of the authors FR, KB, LCD, and OK indicates that *I. inopinatus* is endemic throughout Germany in sympatry with *I. ricinus*. To continue to use historical locations and because the majority of recent studies in Europe have not yet differentiated between *I. ricinus* and *I. inopinatus*, the two species are combined herein and referred to as the *I. ricinus/inopinatus* species complex. A separate map for *I. inopinatus* was therefore not compiled.

### *Ixodes (Pholeoixodes) lividus* Koch

The nest-dwelling bird parasite *I. lividus* typically infests sand martins (*Riparia riparia*) and house martins (*Delichon urbicum*). Its global distribution is between 9.5° W–145° E and 34–72° N (Kolonin 2009). In Germany, only a few ticks were found on sand martins, comprising the following numbers of locations: 4 Müller et al. (1975), 1 Walter et al. (1979a), 1 Hesse (1985). In a more recent study three birds infested with a total of 15 specimens of *I. lividus* were mentioned (Klaus et al. 2016). Since no locations were given, these could not be mapped. A total of five out of six known locations are depicted in Fig. 1.

### *Ixodes (Ixodes) ricinus* (L.)

The castor bean tick *I. ricinus* is widely distributed in the Western Palearctic. It occurs from Portugal extending to the Volga river in Russia, and from the north of Finland to the Mediterranean countries including Northern Africa (Otranto et al. 2017). Due to climate change, its range has been expanding both northwards (Jaenson et al. 2012) and to higher mountain areas (Materna et al. 2008; Garcia-Vozmediano et al. 2020). In its distribution range, *I. ricinus* is the main vector of pathogens that cause tick-borne encephalitis and Lyme borreliosis, which is why it is the best-studied tick species in Germany. In addition, *I. ricinus* is also by far the most common tick species flagged from lower vegetation and collected from hosts. It occurs throughout Germany. The 862 georeferenced locations from Rubel et al. (2014) have been supplemented by 159 new locations from the literature (see supplements) and the following numbers of new locations from the authors: 195 EMK, 3 KB, and 26 OK. As mentioned above, *I. ricinus* and *I. inopinatus* are combined herein as *I. ricinus/I. inopinatus*. Together with 776 locations provided by Estrada-Peña et al. (2013) and 217 locations from GBIF (2014), a total of 2238 *I. ricinus/inopinatus* species complex locations are known, from which a selection of 782 is depicted in Fig. 11.

### *Ixodes (Pholeoixodes) rugicollis* Schulze and Schlottke

This tick species was reported from the European countries Austria, France, Poland, Romania, and Switzerland, as well as from a single location in Germany (Pfäffle and Petney 2017). Here, georeferenced coordinates have been compiled from two regions where several ticks were collected from feral minks (*Neovison vison*) by Christian (2010), two regions where several ticks were collected from pine martens (*Martes martes*) by Christian (2002) and one location where the tick was collected from a European polecat (*Mustela putorius*) by Kretschmar (2016). A total of five known locations is depicted in Fig. 8.

### *Ixodes (Pomerantzevella) simplex* Neumann

This bat-parasitizing tick species is found in Eurasia, Africa and Australia (Kolonin 2009). In Europe, the distribution of *I. simplex* is widespread in the countries of the western and eastern Mediterranean sea and the Balkans (Burazerović et al. 2015), while it is found only sporadically in Central European countries. It was recorded only from a single place in Germany (Walter and Kock 1985), where two nymphs in Espenschied, Hesse, were found on a hibernating greater mouse-eared bat (*Myotis myotis*). However, the occurrence of *I. simplex* depends largely on the distribution of its major host, the cave-dwelling bat (*Miniopterus schreibersii*). This thermophilic bat species occurs only sporadically in the southwest of the country, which is probably why *I. simplex* has been rarely found in Germany. Thus, only one known location of *I. simplex* is depicted in Fig. 2.

### *Ixodes (Exopalpiger) trianguliceps* Birula

The shrew or vole tick *I. trianguliceps* is generally found in the nests and burrows of its small mammal hosts in the warm temperate and boreal climate zones of Eurasia. The distribution area between 9° W–88° E and 43–70° N extends from Northern Spain to Western Siberia, but *I. trianguliceps* does not occur in the Mediterranean area (Kolonin 2009). The 4 locations of the dataset by Rubel et al. (2014) have been supplemented by the following number of locations: 11 (Walter et al. 1986), 1 (Olbrich and Liebisch 1991), 1 (Kocianová et al. 1993), 3 (Petney et al. 2013), 1 (Obiegala et al. 2015), 11 LCD. A total of 25 out of 32 known locations is depicted in Fig. 7.

### *Ixodes (Ceratixodes) uriae* White

The seabird tick *I. uriae* has the most extensive geographical distribution of all tick species, including all zoogeographic regions (Muñoz-Leal and González-Acuñab 2015). In Germany, Liebisch and Vauk-Hentzelt (1992) found the species on the island of Helgoland. This is the only location in Germany that has been described so far. It is depicted in Fig. 1.

### *Ixodes (Eschatocephalus) vespertilionis* Koch

The bat tick *I. vespertilionis* is widespread in Europe, preferably in more southern latitudes. In Scandinavia and the Baltic States, the tick is completely absent (Hornok 2017). Other reports include Algeria, Turkey, Iran, Russia and China, with a wide variety of bat species as hosts for both immature and adult stages (Bendjeddou et al. 2016). In Germany, it is relatively seldom found because its main hosts, horseshoe bats (*Rhinolophus* spp.), are regarded as being endangered, and their habitats (caves) are restricted (Hornok et al. 2015). One location in Bavaria, where *I. vespertilionis* was collected from a Natterer’s bat (*Myotis nattereri*), was mentioned by Rupp et al. (2004). A total of five known locations from Walter and Kock (1985) is depicted in Fig. 2.

### *Rhipicephalus sanguineus* (Latreille)

What was originally called the brown dog tick *R. sanguineus* is a complex of closely related species called *Rhipicephalus sanguineus* sensu lato (Nava et al. 2015). It is the most common tick found on dogs especially in urban areas around the world (Dantas-Torres and Otranto 2017). It is reported in the tropical and subtropical climatic zones of all continents, within the latitude belt 42° S–46° N (Kolonin 2009). In the Mediterranean area, georeferenced locations of *R. sanguineus* s.l. have been mapped by Estrada-Peña et al. (2013). Accordingly, the natural northern distribution limit of *R. sanguineus* s.l. is currently south of the Alps at 46° N latitude. Locations north of the Alps, e.g. in Germany, can therefore be traced back to introductions on dogs by travelers returning from abroad. But it is also assumed that *R. sanguineus* s.l. can survive the German winter indoors. The following numbers of locations were digitized from two historical studies: 24 Gothe and Hamel (1973), 6 Hoffmann (1981). Another study reported *R. sanguineus* s.l. on 22 dogs, but without information on the locations. In 16 homes there was a massive occurrence of the brown dog tick, in five cases also humans were attacked (Dongus et al. 1995). A location verified by HD in 2017 indicates that *R. sanguineus* s.l. is still present in Berlin. It is noteworthy that the mentioned findings do not reflect geographic distribution of an established population but are the results of sporadic findings. As a rule, such outbreaks have been eliminated by pest controllers. Despite the frequent occurrence of the tick, only 19 out of 31 known locations could be mapped in Fig. 6.

## Conclusions

A new compilation of tick maps, referred to as atlas of ticks in Germany, was presented here. Although only tick locations published in the scientific literature and from the authors’ collections were used, it cannot be ruled out that some misidentified ticks were also mapped. A comparative test of identification of ticks occurring in Western Europe and Northern Africa carried out by Estrada-Peña et al. (2017a) showed that a not negligible proportion of ticks were misidentified by the laboratories involved. Accordingly, the proportion of misclassification of the most common tick in Germany, *I. ricinus*, was around 5% and those of the second most common ticks *D. marginatus* and *D. reticulatus* was 7%. Significantly higher proportions of misclassification were found for tick species that are rare in Germany.

There are still considerable gaps in our knowledge of the occurrence of several tick species in Germany. This is especially true for the bat ticks *C. vespertilionis*, *I. ariadnae*, *I. simplex*, and *I. vespertilionis*. For *I. ariadnae* and *I. simplex* only a single observation for either of them was documented. The remarkably high number of 32 locations of *C. vespertilionis* are almost all in the northwest of Germany (Fig. 2). With one exception, there are no documented locations from the southern or eastern federal states and the latest publication from Germany was already 10 years ago. The same applies to the pigeon tick *A. reflexus*. The question is how present this tick species is still in Germany. That there are no recent reports can also mean that the abundance of the pigeon tick is much lower now than 20–30 years ago described by Dautel et al. (1991, 1999). At least 2 recent *A. reflexus* locations determined by HD were mapped here. In addition, there are no recent publications on the occurrence of the brown dog tick *R. sanguineus* s.l. in Germany. However, an ongoing citizen science project may update our knowledge on the occurrence of this tick species complex (Fachet et al. 2019). Accordingly, *R. sanguineus* s.l. was found at 10 locations (not mapped) in 2019. This shows the potential of citizen science projects to expand our knowledge of the spread of ticks, which also resulted in alternative distribution maps for *D. marginatus* and *D. reticulatus* (Drehmann et al. 2020).

Another positive development concerns our knowledge of the distribution of *I. frontalis*. One decade after the first German record of *I. frontalis* flagged from vegetation was described (Schorn et al. 2011), another study showed that this tick species is widespread in four federal states and was also found on several dead birds collected all over Germany (Drehmann et al. 2019). These two studies and 23 new records by LCD and OK have made a major contribution to the fact that a map is now available for the whole of Germany, in which 63 *I. frontalis* locations are shown (Fig. 8). Publications like the one by Agoulon et al. (2019) and Plantard et al. (2021), describing the habitat structure in which high numbers of *I. frontalis* larvae were collected from the vegetation, will also help to improve our knowledge of this up to now little-known tick species.

At this point it must be mentioned that rarely detected tick species whose distribution area is definitely not in Central Europe are not taken into account here. This includes the single record of *Ixodes ventalloi* (Beichel et al. 1996; Petney et al. 1996) and the redetermination of *I. canisuga* collected from red foxes in Thuringia (Meyer-Kayser et al. 2012), that led to the first description of *I. kaiseri* in Germany (Hornok et al. 2017). Currently, however, no exact location of *I. kaiseri* in Germany is known and the summary of descriptions and redescriptions of *I. kaiseri* shows that there are different opinions about the taxonomic status of this tick species (Guglielmone et al. 2020). During the preparation of the present manuscript, a study appeared on the morphology of *Pholeoixodes* species with a pictorial key based on molecularly identified *I. canisuga*, *I. hexagonus* and *I. kaiseri* (Hornok et al. 2021). These findings will help update the distribution maps of the corresponding tick species.

Finally, the results of the tick mapping for the individual federal states of Germany are summarized. Table 1 shows that only *I. ricinus* has been documented in all 16 federal states. The occurrence of *I. inopinatus* reported from 13 federal states has been marked with a circle because it is not shown in a separate map. In the absence of genetic analysis in older studies, morphologically determined *I. ricinus* would have included some *I. inopinatus*. As already mentioned, about 1–7% of *I. ricinus* collected can be assigned to this newly described species (Chitimia-Dobler et al. 2018; Hauck et al. 2019), which might be distributed throughout Germany. Further occurrences of ticks marked with a circle, for which no coordinates are provided and which are therefore not shown on a map, concern *C. vespertilionis* and *I. vespertilionis* in Bavaria (Rupp et al. 2004). The ornate tick *D. reticulatus*, which was found in 15 federal states, occupies the second place in the frequency distribution. Individual *D. reticulatus* found in the federal states of Bremen and Schleswig-Holstein (Drehmann et al. 2020) need confirmation and are therefore not part of the data set presented. These tick locations have again been marked with a circle in Table 1. Only in the federal state of Hamburg *D. reticulatus* was not detected, but this does not mean that the tick species does not occur there. The common tick species *I. hexagonus*, *I. frontalis*, *I. canisuga*, and *A. reflexus* may also occur in all federal states of Germany, although this has not yet been documented. At the other end of the frequency distribution there are six tick species that have only been detected in one federal state.

Table 1 also shows how many tick species have been found in each federal state, according to which Hesse leads with 18 documented tick species, followed by Lower Saxony with 16, and Baden-Wuerttemberg and North Rhine-Westphalia with 14 documented tick species each. These statistics can also be used for the targeted selection of federal states without having much knowledge of the tick fauna in order to initiate new field studies.

## Supplementary Information

Below is the link to the electronic supplementary material.

Supplementary material 1 (xlsx 130 KB)

## Data Availability

Dataset of georeferenced tick locations.

## References

[CR1] Agoulon A, Hoch T, Heylen D, Chalvet-Monfray K, Plantard O (2019). Unravelling he phenology of *Ixodes frontalis*, a common but understudied tick species in Europe. Ticks Tick Borne Dis.

[CR2] Aiello-Lammens ME, Boria RA, Radosavljevic A, Vilela B, Anderson RP (2015). spThin: an R package for spatial thinning of species occurrence records for use in ecological niche models. Ecography.

[CR3] Aiello-Lammens ME, Boria RA, Radosavljevic A, Vilela B, Anderson RP, Bjornson R, Weston S (2019) spThin: Functions for spatial thinning of species occurrence records for use in ecological models

[CR4] Apanaskevich DA, Horak IG (2008). The genus Hyalomma Koch, 1844: v. re-evaluation of the taxonomic rank of taxa comprising the H. (Euhyalomma) marginatum Koch complex of species (Acari: Ixodidae) with redescription of all parasitic stages and notes on biology. Internat J Acarol.

[CR5] Beichel E, Petney TN, Hassler D, Brückner M, Maiwald M (1996). Tick infestation patterns and prevalence of Borrelia burgdorferi in ticks collected at a veterinary clinic in Germany. Vet Parasitol.

[CR6] Bendjeddou ML, Bouslama Z, Amr ZS, Banihani R (2016). Infestation and seasonal activity of *Ixodes vespertilionis* Koch, 1844 (Acari: Ixodidae) on the Maghreb mouse-eared bat, Myotis punicus Felten, 1977, in northeastern Algeria. J Vector Ecol.

[CR7] Burazerović J, Cakić S, Mihaljica D, Sukara R, Ćirović D, Tomanović S (2015). Ticks (Acari: Argasidae, Ixodidae) parasitizing bats in the central Balkans. Exp Appl Acarol.

[CR8] Bursali A, Keskin A, Tekin S (2012). A review of the ticks (Acari: Ixodida) of Turkey: species diversity, hosts and geographical distribution. Exp Appl Acarol.

[CR9] Chen Z, Yang X, Bu F, Yang X, Yang X, Liu J (2010). Ticks (Acari: Ixodoidea: Argasidae, Ixodidae) of China. Exp Appl Acarol.

[CR10] Chitimia-Dobler L, Nava S, Bestehorn M, Dobler G, Wölfel S (2016). First detection of Hyalomma rufipes in Germany. Ticks Tick Borne Dis.

[CR11] Chitimia-Dobler L, Rieß R, Kahl O, Wölfel S, Dobler G, Nava S, Estrada-Peña A (2018). Ixodes inopinatus—occurring also outside the Mediterranean region. Ticks Tick-Borne Dis.

[CR12] Chitimia-Dobler L, Schaper S, Rieß R, Bitterwolf K, Frangoulidis D, Bestehorn M, Springer A, Oehme R, Drehmann M, Lindau A, Mackenstedt U, Strube C, Dobler G (2019). Imported Hyalomma ticks in Germany in 2018. Parasit Vectors.

[CR13] Christian A (2002). Ticks (Ixodida) parasitising the pine marten (*Martes martes*) in the Mecklenburg region. Abh Ber Naturkundemus Görlitz.

[CR14] Christian A (2010). Tick infestation (Ixodes) on feral mink (Neovison vison) in central Germany. Soil Org.

[CR15] Christian A (2012). Tick infestation (Ixodes) on the Eurasian otter (Lutra lutra)—a longterm study. Soil Org.

[CR16] Cornely M, Schultz U (1992). On the tick fauna of Eastern Germany. Angew Parasitol.

[CR17] Dantas-Torres F, Otranto D (2017) *Rhipicephalus sanguineus* s.l. (Latreille, 1806). In: Estrada-Peña A, Mihalca AD, Petney TN (eds) Ticks of Europe and North Africa. A Guide to Species Identification, Springer, Cham, pp 323–327, 10.1007/978-3-319-63760-0

[CR18] Dautel H, Kahl O, Knülle W (1991). The soft tick *Argas reflexus* (F.) (Acari, Argasidae) in urban environments and its medical significance in Berlin (West). J Appl Ent.

[CR19] Dautel H, Scheurer S, Kahl O (1999). The pigeon tick (*Argas reflexus*): its biology, ecology, and epidemiological aspects. Zentbl Bakteriol.

[CR20] Dongus H, Zahler M, Gothe R (1995). The brown dog tick, *Rhipicephalus sanguineus* (Ixodidae), in Germany: an epidemiologic study and control measures. Berl Munch Tierarztl Wochenschr.

[CR21] Drehmann M, Chitimia-Dobler L, Lindau A, Frank A, Mai S, Fachet K, Hauck D, Knoll S, Strube C, Lühken R, Fischer D, Ziegler L, Mackenstedt U (2019). Ixodes frontalis: a neglected but ubiquitous tick species in Germany. Exp Appl Acarol.

[CR22] Drehmann M, Springer A, Lindau A, Fachet K, Mai S, Thoma D, Schneider CR, Chitimia-Dobler L, Bröker M, Dobler G, Mackenstedt U, Strube C (2020). The spatial distribution of Dermacentor ticks (Ixodidae) in Germany-evidence of a continuing spread of *Dermacentor reticulatus*. Front Vet Sci.

[CR23] Dries R (2013) Study of the tick-density and community structure in the Rhine Plain near Offenburg. Bachelor thesis, Karlsruhe Institute for Technology, Germany

[CR24] Estrada-Peña A (2017) *Ixodes inopinatus* Estrada-Peña, Nava and Petney, 2014. In: Estrada-Peña A, Mihalca AD, Petney TN (eds) Ticks of Europe and North Africa. A Guide to Species Identification, Springer, Cham, pp 203–206, 10.1007/978-3-319-63760-0

[CR25] Estrada-Peña A, Farkas R, Jaenson TGT, Koenen F, Madder M, Pascucci I, Salman M, Tarrés-Call J, Jongejan F (2013). Association of environmental traits with the geographic ranges of ticks (Acari: Ixodidae) of medical and veterinary importance in the western Palearctic. A digital data set. Exp Appl Acarol.

[CR26] Estrada-Peña A, Nava S, Petney T (2014). Description of all the stages of *Ixodes inopinatus* n. sp. (Acari: Ixodidae). Ticks Tick-Borne Dis.

[CR27] Estrada-Peña A, D’Amico G, Palomar AM, Dupraz M, Fonville M, Heylen D, Habela MA, Hornok S, Lempereur L, Madder M, Núncio MS, Otranto D, Pfäffle M, Plantard O, Santos-Silva MM, Sprong H, Vatansever Z, Vial L, Mihalca AD (2017). A comparative test of ixodid tick identification by a network of European researchers. Ticks Tick-Borne Dis.

[CR28] Estrada-Peña A, Mihalca AD, Petney TN (2017). Ticks of Europe and North Africa. A guide to species identification.

[CR29] Fachet K, Lindau A, Drehmann M, Mackenstedt U (2019) Die Braune Hundezecke–Aktuelle Studien zu *Rhipicephalus sanguineus* s.l. in Deutschland (in German). Proc. 5th South German Tick Congress, University of Hohenheim, Germany, 2–4 March 2020 https://www.zeckenkongress.de/programm

[CR30] Faulde MK, Rutenfranz M, Hepke J, Rogge M, Görner A, Keth A (2014). Human tick infestation pattern, tick-bite rate, and associated Borrelia burgdorferi s.l. infection risk during occupational tick exposure at the Seedorf military training area, northwestern Germany. Ticks Tick Borne Dis.

[CR31] Fuhrmans R, Manske U (1983). Die Taubenzecke, Argas reflexus, als Parasit des Menschen. Akt Dermatol.

[CR32] Garcia-Vozmediano A, Krawczyk AI, Sprong H, Rossi L, Ramassa E, Tomassone L (2020). Ticks climb the mountains: Ixodid tick infestation and infection by tick-borne pathogens in the Western Alps. Ticks Tick Borne Dis.

[CR33] GBIF (2014) Global Biodiversity Information Facility. Free and open access to biodiversity data. http://www.gbif.org, accessed 30 Jul 2014

[CR34] Geurden T, Becskei C, Six RH, Maeder S, Latrofa MS, Otranto D, Farkas R (2018). Detection of tick-borne pathogens in ticks from dogs and cats in different European countries. Ticks Tick Borne Dis.

[CR35] Gilgenast M (2013) Vergleich der Abundanz von *D. marginatus* auf Schafen am Bienwald (Südpfalz) in den Jahren 2011–2013 (in German). Thesis, Karlsruhe Institute for Technology, Germany

[CR36] Gothe R, Hamel HD (1973). Epizootics of Rhipicephalus sanguineus (Latreille, 1806) in Germany. Zbl Vet Med B.

[CR37] Graef KH (2000). Lederzecken der Art Argas reflexus als Ektoparasiten bei der Schleiereule (Tyto alba). Orn Jh Bad-Württ.

[CR38] Guglielmone AA, Robbins RG, Apanaskevich DA, Petney TN, Estrada-Peña A, Horak IG (2014) The Hard Ticks of the World (Acari: Ixodida: Ixodidae). Springer, Dordrecht, p 738, 10.1007/978-94-007-7497-1

[CR39] Guglielmone AA, Petney TN, Robbins RG (2020) Ixodidae (Acari: Ixodoidea): descriptions and redescriptions of all known species from 1758 to December 31, 2019. Zootaxa 4871:1–322, 10.11646/zootaxa.4871.1.1 10.11646/zootaxa.4871.1.1 33311340

[CR40] Haitlinger R, Walter G (1997). Data relating to the distribution and host-specificity of bat-infesting mites (Acari, Mesostigmata, Prostigmata, Astigmata) in Germany. Drosera.

[CR41] Hauck D, Springer A, Pachnicke S, Schunack B, Fingerle V, Strube C (2019). Ixodes inopinatus in northern Germany: occurrence and potential vector role for Borrelia spp., Rickettsia spp., and Anaplasma phagocytophilum in comparison with Ixodes ricinus. Parasitol Res.

[CR42] Hesse GH (1985). Brutverluste von Uferschwalben (Riparia riparia) durch massive Parasitierung nestbewohnender Flöhe und Zecken. Orn Mitt.

[CR43] Hoffmann G (1981). Die Braune Hundezecke (Rhipicephalus sanguineus L.) in Berlin (West) (in German). Bundesgesundheitsblatt.

[CR44] Hofmeester TR, van der Lei PB, Leeuwen AD, Sprong H, van Wieren SE (2016). New foci of Haemaphysalis punctata and Dermacentor reticulatus in the Netherlands. Ticks Tick Borne Dis.

[CR45] Hoogstraal H (1956) African Ixodoidea I. Ticks of the Sudan. Department of the Navy, Bureau of Medicine and Surgery, Washington DC, 10.5962/bhl.title.6870

[CR46] Hornok S (2017) *Ixodes vespertilionis* Koch, 1844. In: Estrada-Peña A, Mihalca AD, Petney TN (eds) Ticks of Europe and North Africa. A Guide to Species Identification, Springer, Cham, pp 97–101, 10.1007/978-3-319-63760-0

[CR47] Hornok S, Takács N, Szoke K, Kunz B (2015). First record of *Ixodes ariadnae* in Germany. Acta Vet Hungarica.

[CR48] Hornok S, Sándor AD, Beck R, Farkas R, Beati L, Kontschán J, Takács N, Földvári G, Silaghi C, Meyer-Kayser E, Hodžić A, Tomanović S, Swaid A, Wall R, Estrada-Peña A, Duscher GG, Plantard O (2017) Contributions to the phylogeny of *Ixodes (Pholeoixodes) canisuga*, *I. (Ph.) kaiseri*, *I. (Ph.) hexagonus* and a simple pictorial key for the identification of their females. Parasit Vectors 10:545, 10.1186/s13071-017-2424-x 10.1186/s13071-017-2424-x PMC5670724 29100530

[CR49] Hornok S, Kováts D, Horváth G, Kontschán J, Farkas R (2020). Checklist of the hard tick (Acari: Ixodidae) fauna of Hungary with emphasis on host-associations and the emergence of Rhipicephalus sanguineus. Exp Appl Acarol.

[CR50] Hornok S, Meyer-Kayser E, Kontschán J, Takács N, Plantard O, Cullen S, Gaughran A, Szekeres S, Majoros G, Beck R, Boldogh SA, Horváth G, Kutasi C, Sándor AD (2021) Morphology of *Pholeoixodes* species associated with carnivores in the western Palearctic: Pictorial key based on molecularly identified *Ixodes (Ph.) canisuga, I. (Ph.) hexagonus and I. (Ph.) kaiseri* males, nymphs and larvae. Ticks Tick Borne Dis 12:101715, 10.1016/j.ttbdis.2021.101715 10.1016/j.ttbdis.2021.101715 33819744

[CR51] Hubálek Z, Sedláček P, Estrada-Peña A, Vojtíšek J, Rudolf I (2020). First record of Hyalomma rufipes in the Czech Republic, with a review of relevant cases in other parts of Europe. Ticks Tick-Borne Dis.

[CR52] Hudde H, Walter G (1988). Verbreitung und Wirtswahl der Vogelzecke Ixodes arboricola (Ixodoidea, Ixodidae) in der Bundesrepublik Deutschland. Die Vogelwarte.

[CR53] Jaenson TGT, Jaenson DGE, Eisen L, Petersson E, Lindgren E (2012). Changes in the geographical distribution and abundance of the tick Ixodes ricinus during the past 30 years in Sweden. Parasit Vectors.

[CR54] Jameson LJ, Medlock JM (2011). Tick surveillance in Great Britain. Vector Borne Zoonotic Dis.

[CR55] Kahl O, Janetzki C, Gray J, Stein J, Bauch R (1992). Tick infection rates with Borrelia: Ixodes ricinus versus Haemaphysalis concinna and Dermacentor reticulatus in two locations in eastern Germany. Med Vet Entomol.

[CR56] Kahl O, Bulling I, Chitimia-Dobler L (2019). Questing Ixodes frontalis larvae in a forest close to Berlin (Germany) in November 2018. Ticks Tick Borne Dis.

[CR57] Kampen H, Poltz W, Hartelt K, Wölfel R, Faulde M (2007). Detection of a questing Hyalomma marginatum marginatum adult female (Acari, Ixodidae) in southern Germany. Exp Appl Acarol.

[CR58] Kimmig P (2010). Q-Fieber - Eine Infektion mit komplexer Epidemiologie. Denisia.

[CR59] Klaus C, Gethmann J, Hoffmann B, Ziegler U, Heller M, Beer M (2016). Tick infestation in birds and prevalence of pathogens in ticks collected from different places in Germany. Parasitol Res.

[CR60] Kocianová E, Kozuch O, Bakoss P, Rehácek J, Kovácová E (1993). The prevalence of small terrestrial mammals infected with tick-borne encephalitis virus and leptospirae in the foothills of the southern Bavarian forest, Germany. Appl Parasitol.

[CR61] Kolonin GV (2009) Fauna of Ixodid Ticks of the World (Acari, Ixodidae). Online publication no longer available. http://www.kolonin.org accessed on 10 June 2014

[CR62] Kretschmar FM (2016) Die Parasiten des Europäischen Iltisses *Mustela putorius* Linnaeus, 1758 in Deutschland (in German). Doctoral thesis, Univ. Munich, 194pp

[CR63] Kulik IL, Vinokurova NS (1982). Distribution area of Dermacentor marginatus ticks in the USSR. Med Parazitol (Mosk).

[CR64] Liebisch A, Rahman MS (1976). Occurrence and ecology of some tick species (Ixodidae) with medical and veterinary importance in Germany. J Appl Entomol.

[CR65] Liebisch A, Vauk-Hentzelt E (1992). The first record of the tick species Ixodes (Ceratoxides) uriae White, 1852 in Germany. Int J Med Microbiol Hyg.

[CR66] Liebisch A, Walter G (1986). Ticks of domestic and wild animals in Germany: on the occurrence and biology of the hedgehog tick (Ixodes hexgonus) and the fox tick (Ixodes canisuga). Dtsch tierärztl Wschr.

[CR67] Mans BJ, Kelava S, Pienaar R, Featherston J, de Castro MH, Quetglas J, Reeves WK, Durden LA, Miller MM, Laverty TM, Shao R, Takano A, Kawabata H, Moustafa MAM, Nakao R, Matsuno K, Greay TL, Evasco KL, Barker D, Barker SC (2021) Nuclear (18S-28S rRNA) and mitochondrial genome markers of *Carios (Carios) vespertilionis (Argasidae)* support Carios Latreille, 1796 as a lineage embedded in the Ornithodorinae: re-classification of the *Carios* sensu Klompen and Oliver (1993) clade into its respective subgenera. Ticks Tick Borne Dis 12:101688, https://doi.org/10.1016/j.ttbdis.2021.101688 10.1016/j.ttbdis.2021.101688 33652332

[CR68] Materna J, Daniel M, Metelka L, Harcarik J (2008) The vertical distribution, density and the development of the tick *Ixodes ricinus* in mountain areas influenced by climate changes (The Krkonose Mts., Czech Republic). Int J Med Microbiol 298(S1):25–37, 10.1016/j.ijmm.2008.05.004

[CR69] Mayer A, Madel W (1950) Beobachtungen über das Auftreten und die Bekämpfung von Taubenzecken (*Argas reflexus* F.) (in German). Desinf Schädlingsbekämpf 41(B):197–199

[CR70] Metz K (1911). Argas reflexus, die Taubenzecke. Monatshefte prakt Tierheilkd.

[CR71] Meyer-Kayser E, Hoffmann L, Silaghi C, Passos L, Mahling M, Pfister K (2011). Tick infestation of foxes in Thuringia with special focus on foxes with scabies. Wien Tierarztl Monatsschr.

[CR72] Meyer-Kayser E, Hoffmann L, Silaghi C, Pfister K, Mahling M, Passos LMF (2012). Dynamics of tick infestations in foxes in Thuringia, Germany. Ticks Tick Borne Dis.

[CR73] Müller J, Ciupa W, Seelig KJ (1975). Zum Vorkommen von Ixodes lividus Koch (syn. I. plumbeus Leach) auf Uferschwalben, Riparia riparia (L.), im Kreis Staßfurt. Hercynia N F.

[CR74] Muñoz-Leal S, González-Acuñab D (2015). The tick Ixodes uriae (Acari: Ixodidae): Hosts, geographical distribution, and vector roles. Ticks Tick Borne Dis.

[CR75] Nauke T (2007) *Dermacentor reticulatus* in Germany and the spread of canine babesiosis. Proc. 2nd CVBD Symposium, Mazara del Vallo, Sicily, 24–28

[CR76] Nava S, Estrada-Peña A, Petney T, Beati L, Labruna MB, Szabó MPJ, Venzal JM, Mastropaolo M, Mangold AJ, Guglielmone AA (2015). The taxonomic status of Rhipicephalus sanguineus (Latreille, 1806). Vet Parasitol.

[CR77] Obiegala A, Pfeffer M, Pfister K, Karnath C, Silaghi C (2015). Molecular examinations of Babesia microti in rodents and rodent-attached ticks from urban and sylvatic habitats in Germany. Ticks Tick Borne Dis.

[CR78] Obsomer V, Wirtgen M, Linden A, Claerebout E, Heyman P, Heylen D, Madder M, Maris J, Lebrun M, Tack W, Lempereur L, Hance T, Van Impe G (2013). Spatial disaggregation of tick occurrence and ecology at a local scale as a preliminary step for spatial surveillance of tick-borne diseases: general framework and health implications in Belgium. Parasit Vectors.

[CR79] Oehme R, Bestehorn M, Wölfel S, Chitimia-Dobler L (2017) *Hyalomma marginatum* in Tübingen, Germany. Syst Appl Acarol 22:1–6, 10.11158/saa.22.1.1

[CR80] Olbrich S, Liebisch A (1991). Epidemiological studies of the infection of ticks with borreliosis agents in small mammals from North Germany. Dtsch tierärztl Wschr.

[CR81] Otranto D, Dantas-Torres F, Santos-Silva MM (2017) *Ixodes ricinus* (Linnaeus, 1758). In: Estrada-Peña A, Mihalca AD, Petney TN (eds) Ticks of Europe and North Africa. A Guide to Species Identification, Springer, Cham, pp 189–195, 10.1007/978-3-319-63760-0

[CR82] Petney T, Pfäffle M, Littwin N, Norra S, Böhnke D, Hogewind F, Gebhardt R, Oehme R, Sebastian P, Steidle J, Kahl O, Dautel H (2013) Untersuchung der Ökologie von Zecken als Überträger von Krankheitserregern in Baden-Württemberg in Bezug auf Habitat, Landnutzung, Wirtstiere und Klima (in German). Project report BWPLUS, Karlsruher Institute of Technology, p 13

[CR83] Petney TN, Beichel E, Maiwald M, Hassler D (1996). Ixodes ventalloi: a new tick record for Germany. Appl Parasitol.

[CR84] Petney TN, Pfäffle MP, Skuballa JD (2012) An annotated checklist of the ticks (Acari: Ixodida) of Germany. System Appl Acarol 17:115–170, 10.11158/saa.17.2.2

[CR85] Petney TN, Moser E, Littwin N, Pfäffle M, Muders SV, Taraschewski H (2015) Additions to the ’Annotated Checklist of the Ticks of Germany’: *Ixodes acuminatus* and *Ixodes inopinatus*. Syst Appl Acarol 20:221–224, 10.11158/saa.20.2.9

[CR86] Petney TN, Jaenson TGT, Pfäffle MP (2017) *Argas vespertilionis* (Latreille, 1796). In: Estrada-Peña A, Mihalca AD, Petney TN (eds) Ticks of Europe and North Africa. A Guide to Species Identification, Springer, Cham, pp 33–36, 10.1007/978-3-319-63760-0

[CR87] Pfäffle M, Petney T, Skuballa J, Taraschewski H (2011). Comparative population dynamics of a generalist (Ixodes ricinus) and specialist tick (I. hexagonus) species from European hedgehogs. Exp Appl Acarol.

[CR88] Pfäffle MP, Petney TN (2017) *Ixodes rugicollis* Schulze and Schlottke, 1929. In: Estrada-Peña A, Mihalca AD, Petney TN (eds) Ticks of Europe and North Africa. A Guide to Species Identification, Springer, Cham, pp 163–165, 10.1007/978-3-319-63760-0

[CR89] Pfäffle MP, Madder M, Santos-Silva MM, Petney TN (2017) *Ixodes frontalis* (Panzer, 1798). In: Estrada-Peña A, Mihalca AD, Petney TN (eds) Ticks of Europe and North Africa. A Guide to Species Identification, Springer, Cham, pp 91–96, 10.1007/978-3-319-63760-0

[CR90] Plantard O, Hoch T, Daveu R, Rispe C, Stachurski F, Boué F, Poux V, Cebe N, Verheyden H, René-Martellet M, Chalvet-Monfray K, Cafiso A, Olivieri E, Moutailler S, Pollet T, Agoulon A (2021). Where to find questing Ixodes frontalis ticks? Under bamboo bushes!. Ticks Tick Borne Dis.

[CR91] Pluta S (2011) Epidemiologie von *Coxiella burnetii*, *Rickettsia* spp., FSME- und Hantaviren in Süddeutschland unter Berücksichtigung klimatischer Veränderungen (in German). Doctoral thesis, Univ. Hohenheim, Germany, p 255

[CR92] Pluta S, Hartelt K, Oehme R, Mackenstedt U, Kimmig P (2010). Prevalence of Coxiella burnetii and Rickettsia spp. in ticks and rodents in southern Germany. Ticks Tick Borne Dis.

[CR93] R Development Core Team (2019) R: A Language and Environment for Statistical Computing, Version 3.6.2. R Foundation for Statistical Computing, Vienna, Austria http://www.R-project.org

[CR94] Rubel F, Brugger K, Monazahian M, Habedank B, Dautel H, Leverenz S, Kahl O (2014). The first German map of georeferenced ixodid tick locations. Parasit Vectors.

[CR95] Rubel F, Brugger K, Pfeffer M, Chitimia-Dobler L, Didyk YM, Leverenz S, Dautel H, Kahl O (2016). Geographical distribution of Dermacentor marginatus and Dermacentor reticulatus in Europe. Ticks Tick Borne Dis.

[CR96] Rubel F, Brugger K, Walter M, Vogelgesang JR, Didyk YM, Fu S, Kahl O (2018). Geographical distribution, climate adaptation and vector competence of the Eurasian hard tick Haemaphysalis concinna. Ticks Tick Borne Dis.

[CR97] Rubel F, Brugger K, Belova OA, Kholodilov IS, Didyk YM, Kurzrock L, García-Pérez AL, Kahl O (2020). Vectors of disease at the northern distribution limit of the genus Dermacentor in Eurasia: D. reticulatus and D. silvarum. Exp Appl Acarol.

[CR98] Rudolf I, Kejíková R, Vojtíšek J, Mendel J, Peňázziová K, Hubálek Z, Šikutová S, Estrada-Peña A (2021). Probable overwintering of adult Hyalomma rufipes in Central Europe. Ticks Tick Borne Dis.

[CR99] Rumer L, Graser E, Hillebrand T, Talaska T, Dautel H, Mediannikov O, Roy-Chowdhury P, Sheshukova O, Donoso Mantke O, Niedrig M (2011). Rickettsia aeschlimannii in Hyalomma marginatum ticks, Germany. Emerg Inf Dis.

[CR100] Rupp D, Zahn A, Ludwig P (2004). Actual records of bat ectoparasites in Bavaria (Germany). Spixiana.

[CR101] Sándor AD (2017) *Ixodes canisuga* Johnston, 1849. In: Estrada-Peña A, Mihalca AD, Petney TN (eds) Ticks of Europe and North Africa. A Guide to Species Identification, Springer, Cham, pp 137–141, 10.1007/978-3-319-63760-0

[CR102] Santos-Silva MM, Beati L, Santos AS, Sousa RD, Nuncio MS, Melo P, Santos-Reis M, Fonseca C, Formosinho P, Vilela C, Bacellar F (2011). The hard-tick fauna of mainland Portugal (Acari: Ixodidae): an update on geographical distribution and known associations with hosts and pathogens. Exp Appl Acarol.

[CR103] Scheffler I, Hiller A (2010). Zur Ektoparasitenfauna der Fledermäuse in Niedersachsen: Neue Funde am Iberg bei Bad Grund. Nyctalus.

[CR104] Schorn S, Schöl H, Pfister K, Silaghi C (2011). First record of Ixodes frontalis collected by flagging in Germany. Ticks Tick Borne Dis.

[CR105] Schreiber C, Krücken J, Beck S, Maaz D, Pachnicke S, Krieger K, Gross M, Kohn B, Samson-Himmelstjerna G (2014). Pathogens in ticks collected from dogs in Berlin/Brandenburg, Germany. Parasit Vectors.

[CR106] Schulze P (1923). Haemaphysalis concinna Koch (Ixod.) in Brandenburg. Deutsch Ent Zeitschr.

[CR107] Silaghi C, Weis L, Pfister K (2020). Dermacentor reticulatus and Babesia canis in Bavaria (Germany)—a georeferenced field study with digital habitat characterization. Pathogens.

[CR108] Speck S, Perseke L, Petney T, Skuballa J, Pfäffle M, Taraschewski H, Bunnell T, Essbauer S, Dobler G (2013). Detection of Rickettsia helvetica in ticks collected from European hedgehogs (Erinaceus europaeus, Linnaeus, 1758). Ticks Tick Borne Dis.

[CR109] Talaska T, Horitz B, Faulde M (2010). Reliktzecken in ungewöhnlichen Biotopen - Haemaphysalis concinna in Ost-Brandenburg. Brandenburgisches Ärzteblatt.

[CR110] Teng KF (1982). The geographic distribution of the genus Dermacentor in China. Sinozoologica.

[CR111] Toma L, Mancuso E, d’Alessio SG, Menegon M, Spina F, Pascucci I, Monaco F, Goffredo M, Luca MD (2021). Tick species from Africa by migratory birds: a 3-year study in Italy. Exp Appl Acarol.

[CR112] Walter G (1980). Untersuchungen zur Zeckenfauna der Kleinsäuger des Naturschutzgebietes Hagenburger Moor. Beitr Naturk Niedersachsen.

[CR113] Walter G (1985) Koprologische Untersuchungen – eine zeitgemäße Methode zur Erfassung der Ektoparasitenfauna der Fledermäuse (in German). Drosera 85:29–34

[CR114] Walter G, Kock D (1985). Records of Ixodes vespertilionis, I. simplex and Argas vespertilionis (Ixodoidea: Ixodidae, Argasidae) from German bats (Chiroptera). Z Parasitenkd.

[CR115] Walter G, Rackow W (2007). Außergewöhnlich hoher Befall einer Nordfledermaus, Eptesicus nilssonii, mit der Lederzecke, Argas vespertilionis (Argasidae). Nyctalus.

[CR116] Walter G, Liebisch A, Streichert J (1979). Untersuchungen zur Biologie und Verbreitung von Zecken (Ixodoidea, Ixodidae) in Norddeutschland. I. Die Vogelzecken Ixodes lividus (C. L. Koch 1844) und Ixodes arboricola (Schulze & Schlottke 1929). Z Angew Zool.

[CR117] Walter G, Liebisch A, Vauk C (1979). Untersuchungen zur Biologie und Verbreitung von Zecken (Ixodoidea, Ixodidae) in Norddeutschland. II. Zecken der Zugvögel auf der Insel Helgoland. Z Angew Zool.

[CR118] Walter G, Kock D, Liebisch A (1986). Beitrag zur Zecken-Fauna der Bundesrepublik Deutschland. Parasitol Senkenbergiana biol.

[CR119] Walter M, Brugger K, Rubel F (2016). The ecological niche of Dermacentor marginatus in Germany. Parasitol Res.

[CR120] Younsi H, Fares W, Cherni S, Dachraoui K, Barhoumi W, Najjar C, Zhioua E (2020). Ixodes inopinatus and Ixodes ricinus (Acari: Ixodidae) are sympatric ticks in North Africa. J Med Entomol.

